# Alternate-locus aware variant calling in whole genome sequencing

**DOI:** 10.1186/s13073-016-0383-z

**Published:** 2016-12-13

**Authors:** Marten Jäger, Max Schubach, Tomasz Zemojtel, Knut Reinert, Deanna M. Church, Peter N. Robinson

**Affiliations:** 1 Institute for Medical and Human Genetics, Charité-Universitätsmedizin Berlin, Augustenburger Platz 1, Berlin, 13353 Germany; 2 Berlin Brandenburg Center for Regenerative Therapies (BCRT), Charité-Universitätsmedizin Berlin, Augustenburger Platz 1, Berlin, 13353 Germany; 3 Institute for Bioinformatics, Department of Mathematics and Computer Science, Freie Universität Berlin, Arnimallee 14, Berlin, 14195 Germany; 4 10x Genomics, 7068 Koll Center Parkway, Suite 401, Pleasanton, 94566 CA USA; 5 The Jackson Laboratory for Genomic Medicine, 10 Discovery Drive, Farmington, 06032 CT USA; 6 Institute for Systems Genomics, University of Connecticut, Farmington, 06032 CT USA

**Keywords:** GRCh38, NGS, WGS, Genome sequencing

## Abstract

**Background:**

The last two human genome assemblies have extended the previous linear golden-path paradigm of the human genome to a graph-like model to better represent regions with a high degree of structural variability. The new model offers opportunities to improve the technical validity of variant calling in whole-genome sequencing (WGS).

**Methods:**

We developed an algorithm that analyzes the patterns of variant calls in the 178 structurally variable regions of the GRCh38 genome assembly, and infers whether a given sample is most likely to contain sequences from the primary assembly, an alternate locus, or their heterozygous combination at each of these 178 regions. We investigate 121 in-house WGS datasets that have been aligned to the GRCh37 and GRCh38 assemblies.

**Results:**

We show that stretches of sequences that are largely but not entirely identical between the primary assembly and an alternate locus can result in multiple variant calls against regions of the primary assembly. In WGS analysis, this results in characteristic and recognizable patterns of variant calls at positions that we term alignable scaffold-discrepant positions (ASDPs). In 121 in-house genomes, on average 51.8±3.8 of the 178 regions were found to correspond best to an alternate locus rather than the primary assembly sequence, and filtering these genomes with our algorithm led to the identification of 7863 variant calls per genome that colocalized with ASDPs. Additionally, we found that 437 of 791 genome-wide association study hits located within one of the regions corresponded to ASDPs.

**Conclusions:**

Our algorithm uses the information contained in the 178 structurally variable regions of the GRCh38 genome assembly to avoid spurious variant calls in cases where samples contain an alternate locus rather than the corresponding segment of the primary assembly. These results suggest the great potential of fully incorporating the resources of graph-like genome assemblies into variant calling, but also underscore the importance of developing computational resources that will allow a full reconstruction of the genotype in personal genomes. Our algorithm is freely available at https://github.com/charite/asdpex.

**Electronic supplementary material:**

The online version of this article (doi:10.1186/s13073-016-0383-z) contains supplementary material, which is available to authorized users.

## Background

The initial assembly of the human genome resulted in a consensus haploid representation of each chromosome that was the best attainable consensus sequence for the human genome, the golden path [1–3]. Variants could then be represented by an annotation to the corresponding position of the golden-path assembly. However, subsequent research showed that large-scale structural variation is more prevalent than previously thought, and that it is not possible to adequately represent genomic regions with substantial structural allelic diversity using a single consensus sequence for the human genome [4].

The Genome Reference Consortium (GRC) introduced a new graph-like assembly model with alternative sequence paths in regions with complex structural variation in the form of additional locus sequences. While the previous genome assembly, GRCh37 (also called hg19), included three regions with nine alternate locus sequences, the GRCh38 assembly, which was released in December 2013, has a total of 178 regions with 261 alternate loci. This offers many opportunities to the genomics and bioinformatics communities to adapt analysis procedures to a more sophisticated model of the human genome, but it also presents substantial technical challenges, since many of the currently used programs for alignment, variant calling, and analysis expect reads and features to have a single location within a haploid assembly model [4]. The (SAM) format for sequence alignments is able to represent reads that align both to chromosomes of the primary assembly (i.e., chr1-22, chrX, chrY, and chrMT) and to an alternate locus, whereby the alignment to the chromosome of the primary assembly is considered to be the representative alignment, and alignments of the same read to non-reference chromosomes, called supplementary alignments, are linked to the representative alignments. However, analogous extensions of the alogous extensions that would link variants called from the same reads to multiple loci are not available.

The characterization of variants in an individual genome is one of the most important tasks in medical genomics, especially in diagnostic settings or in projects that aim to identify novel disease-associated genes. Alternate loci for highly variable regions such as the Major Histocompatibility Complex (MHC) locus may differ at tens of thousands of positions [5], and thus there is a substantial potential to improve the accuracy of variant calls by exploiting the information in the new genome assembly model. In this work, we present an analysis of the implications of the alternate loci for variant calling.

We provide an implementation of the algorithms used in the work under a Creative Commons CC-BY 4.0 license at https://github.com/charite/asdpex.

## Methods

### GRCh38 genome assembly data

Prior to the GRCh37 assembly, the human genome reference sequence was represented as a single consensus sequence referred to as the golden path [1]. Several chromosomal regions display a sufficiently high degree of variability that they cannot be adequately represented by a single sequence [6–8]. For this reason, the GRC began to provide alternate sequences for selected variant regions through the inclusion of alternate locus scaffolds (or alternate loci) starting with the GRCh37 human genome assembly [9].

An alternate locus is a sequence that is an alternate representation of a genomic region in a largely haploid assembly. Thus, alternate loci are provided for genomic regions that show substantial variability in the population and are embedded in an otherwise haploid representation of the genome. For the analysis described in this work, the Genome Reference Consortium build 38 patch 2 assembly of the human genome was used (GRCh38.p2). This build has a total of 178 alternate-locus-containing regions associated with a total of 261 alternate locus scaffolds.

In our work, we refer to segments of the primary assembly unit that are associated with one or more alternate loci as REF-HAP sequences, and we refer to the alternate loci as ALT-HAP sequences. The alternate loci have a number of different patterns of alignment with the REF-HAP sequence (Additional file 1: Supplemental Figures S1–S5 and Additional file 1: Supplemental Table S1).

### Alignments of ALT-HAP and REF-HAP sequences

For each of the 178 alternate-locus-containing regions in the GRCh38.p2 assembly, we performed pairwise alignments between the REF-HAP and each of the alternate loci at the region. Alignments for each of the alternate loci in the alts_accessions_GRCh38.p2 and the various alt_scaffold_placement.txt files were downloaded from the National Center for Biotechnology Information (NCBI) FTP site [10]. The alignments start with the Gap= flag, followed by several blocks consisting of a letter (M, I, or D) and a number (length), where (i) M indicates a matching region between ref and alt loci, potentially with mismatches but without gaps; (ii) I indicates an insertion in the alt scaffold (i.e., additional sequence information in the alt scaffold); and (iii) D indicates a deletion in the alt scaffold (e.g., Gap = M23343 D19 M5 D24 M4 D11 M88 D1 M54 I1 M59...). Manual inspection revealed that parts of these alignments are often suboptimal in the sense that potentially alignable regions are split into multiple small alignment blocks (Additional file 1: Supplemental Figures S6 and S7).

Therefore, we used the following strategy to redefine the sequence alignments for each alignment of an alternate scaffold against the reference. Our method identifies seed sequences as relatively long M blocks (i.e., matches or mismatches with no gaps). In many cases, there are multiple mismatching bases at the very beginning and end of M blocks, and for this reason we remove 5% but not more than 50 nt on both ends of the seed. For each match (M) block in the original alignment, we considered the sequence to be a seed sequence if the trimmed M block was longer than 50 nt (Additional file 1: Supplemental Figure S8).

Some of the general feature format (GFF) files representing the alignments contain a second alignment line. In this case, one finds a large insertion (I) followed by a large deletion (D) in the main alignment; this represents an inversion (see Additional file 1: Supplemental Figure S8). To handle this, we split alignments into blocks defined by the large insertion/deletion boundaries and aligned these blocks separately.

Another relevant issue with the alignments is that they ignore long stretches with Ns in the alternate scaffold sequences, that is, N bases are treated as matches (e.g., KI270905.1, GL000258.2, and GL383571.1). If the stretch of N bases was larger than 10 nt, we split the seed into two separate blocks to either side of the N bases.

The preprocessing steps described above are summarized in Additional file 1: Supplemental Algorithm S1. The output of the preprocessing consists of a series of seeds of 50 nt or longer in length. The seeds were then used for a banded chain alignment [11] using the C++ library SeqAn [12] version 2.0.1. The parameters used for the alignment were match: 5, mismatch: −2, gapextend: 0, gapopen: −20, and anchor bands: 10.

Finally, we note that the alignments between regions and alternate loci begin with identical anchors, such that the beginning and end portions of the alignments are identical. No differences between the two sequences occur in these regions, and the analysis described in the following sections was, thus, limited to the portion of the alignment between the first and the last difference within each alignment (Additional file 1: Supplemental Figure S9).

### Identification of alignable scaffold-discrepant positions

We show that stretches of sequences that are largely but not entirely identical between the primary assembly and an alternate locus can result in multiple variant calls against regions of the primary assembly. We will refer to divergent positions within otherwise similar or identical stretches of alignment as alignable scaffold-discrepant positions (ASDPs); the following text will make our definition more precise.

The alignment resulting from the procedure described in the previous section was taken as the basis for the following algorithm to identify ASDPs. Each position of the alignment was checked in turn for a mismatch or gap, and all such positions were recorded in a VCF file. ASDPs comprise mainly single-nucleotide differences, but insertions and deletions of various sizes are encountered. For the analysis described in this work, we classified insertion or deletion (indel) ASDPs into small-indel ASDPs with a size less than 50 bases or structural variant (SV) ASDPs. This cutoff was chosen since most variant detection tools (e.g., FreeBayes [13] and GATK [14]) only call indels up to this size.

The first-pass analysis described above generates a list of candidate ASDPs (Additional file 1: Supplemental Figure S10). We chose to restrict the final analysis to ASDPs that are located within relatively good segments of the alignment. Therefore, the final list of ASDPs was generated by applying the filter that no ASDP can be located in any 50 base-pair window of the alignment in which there are more than ten discrepant positions. That is, the window is advanced one nucleotide at a time, and if there is any window position at which there are more than ten discrepant positions, then the candidate ASDP is discarded. We will refer to this final list of ASDPs as *high-confidence* ASDPs.

We note that we use the acronym ASDP to refer to a divergent position in the alignment between REF-HAP and ALT-HAP sequences, and not to a called variant; we will show that many variants called in whole-genome sequencing (WGS) overlap with ASDPs, and we will refer to such variants as *ASDP-associated variants*.

### ASDPex

ASDPex, the ASDP extraction algorithm, is designed to analyze individual VCF files with the goal of identifying ASDP-associated variants so that they can be marked or filtered out of downstream analysis pipelines if desired (Additional file 1: Supplemental Figure S11). Additionally, ASDPex calls the most likely combination of haplotypes for each of the 178 genomic regions.

For this purpose, ASDPex scans each of the 178 regions in turn and compares all of the associated alternate loci. For each comparison, all variants called against the reference haplotype are assigned to the set $\mathcal {R}$. All ASDPs associated with the alternate locus are assigned to the set $\mathcal {A}$ (many but not necessarily all of these ASDPs can have corresponding ASDP-associated variants). We define the set of residual variants RV to be the set of all called variants that are not ASDP-associated and all ASDPs that are not called in the sample, which can be expressed as the symmetric set difference $\text {\texttt {RV}}=\mathcal {R}\triangle \mathcal {A}$. For this calculation, we treat an ASDP-associated variant as equivalent to the corresponding ASDP:

$$\begin{array}{*{20}l}{} \text{\texttt{RV}} = \mathcal{R} \triangle \mathcal{A} = (\mathcal{R} \setminus \mathcal{A}) \cup (\mathcal{A} \setminus \mathcal{R}) = \mathcal{R}\cup \mathcal{A} \setminus (\mathcal{R} \cap \mathcal{A}). \end{array} $$

Note that $\mathcal {R} \setminus \mathcal {A}$ is the set of non-ASDP variants called against REF-HAP, and $\mathcal {A} \setminus \mathcal {R}$ is the set of ASDPs associated with ALT-HAP *not* called against REF-HAP (if we assume that ALT-HAP is truly present, then this could be a false negative due to a factor such as poor coverage but our model interprets it as a variant in the REF-HAP sequence). It is easy to see that the number of residual variants is $\left | \text {\texttt {RV}}\right | = \left | \mathcal {R}\right | + \left | \mathcal {A}\right | - 2 \times \left | \mathcal {R}\cap \mathcal {A} \right |$.

The assumption of our algorithm is that the haplotype associated with the lower number of variants is more likely to be present. Thus, if $\left |\mathcal {R}\right |\!>\!\!\left |\text {\texttt {RV}}\right |$, REF-HAP would be associated with more variants than if we assume the presence of ALT-HAP. Therefore, ASDPex infers that ALT-HAP is present.

If on the other hand, $\left |\text {\texttt {RV}}\right |\geq \left |\mathcal {R}\right |$, more or equal variants would be called against ALT-HAP than for REF-HAP, and ASDPex infers that the alternate locus is not present. If the algorithm infers that an alternate locus is present, then it calculates the proportion of variants that correspond to ASDPs that are also homozygous. If this proportion is over a threshold (for the analysis presented here, we chose the threshold to be 90%), then our procedure infers that the ALT-HAP is likely to be present in a homozygous state, otherwise it is heterozygous (Additional file 1: Supplemental Algorithm S2).

Finally, if the region *R* is associated with more than one alternate locus, then we need to decide which, if any, alternate locus is present. To do so, we calculate the number of residual variants *RV* for each alternate locus. The locus with the smallest value for *RV* is the best candidate, and our procedure considers only this locus. We note that our procedure is a heuristic that considers only variants called against the canonical chromosomes in a VCF file resulting from an analysis using the GRCh38 genome assembly.

### Alignment of whole-genome sequencing samples and variant calling

To validate the ASDPs against real data, we used 121 genomes sequenced on an Illumina HiSeq X-Ten system (Macrogen, Seoul, Korea). The reads were aligned to the GRCh37 and GRCh38 genome releases with BWA-MEM (version 0.7.12-r1039) utilizing bwakit (https://github.com/lh3/bwa/tree/master/bwakit). This tool, which can be used to align reads to either the GRCh37 or GRCh38 assembly, trims the reads (trimadap), and aligns the trimmed reads to the reference with BWA-MEM [15]. We run bwa mem (using the run-bwamem script) as follows:



We note that bwa mem aligns reads to the primary assembly and the alternate loci independently, thus avoiding the potential problem that a read that aligns well to a sequence in the primary assembly and another sequence in an alternate locus is given a poor mapping quality. In this work, we used the bwa mem alignments to the alternate loci for visualization, but we note that ASDPex uses only variant calls to the primary assembly and, thus, an alignment performed by any mapper to just the primary assembly could also be used as input to ASDPex.

Finally, samtools [16] was used to sort the alignment and SAMBLASTER [17] to mark duplicates, which resulted in the final alignment. This final alignment was then used to call variants [single nucleotide variants (SNVs) and small indels] using FreeBayes [13]. There was a mean 37-fold coverage.

Variants were normalized using vcflib vcfallelicprimitives (https://github.com/ekg/vcflib--v1.0.0) and vt normalize (https://github.com/atks/vt--v0.57).

### Data sources

The *hs37d5* reference is assembled from the GRCh37 primary assembly, the EBV genome and the decoy contigs as used by 1000 Genome Project [18] phase 3. The *hs38DH* reference contains the primary assembly of GRCh38 plus the ALT contigs and additionally decoy contigs and HLA genes. This assembly is strongly recommended for GRCh38 mapping by the BWA-kit pipeline. The current dbSNP release (b146) was downloaded as a VCF file from the NCBI [10] FTP site for both genome releases. We adopt dbSNP’s definition of a common polymorphism as one with a minor allele of frequency ≥1% and for which two or more founders contribute to that minor allele frequency. All other polymorphic sites in dbSNP are considered rare.

The genomic feature annotations (e.g., exons and coding sequence[CDS]) for RefSeq genes [19] and the genome builds GRCh37.p13 and GRCh38.p2 were downloaded from the NCBI FTP site. Transcript-based functional annotation was performed with Jannovar (version 0.16) [20].

The genome-wide association study GWAS catalog [21] was downloaded on 1 February 2016. It contained 18,130 unique single nucleotide polymorphisms (SNPs) (GWAS hits) with chromosomal coordinates that were significantly associated with a disease or trait at a *p* value of less than 10^−5^.

## Results

**Fig. 1:**
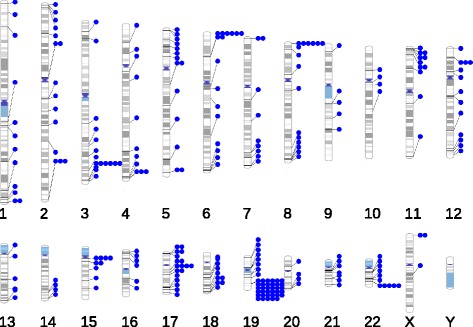
Genomic regions with alternate locus scaffolds (alternate loci). The GRCh38.p2 genome assembly contains 178 genomic regions with one or more alt loci. The figure was produced using PhenoGram [49]

In this work, we explore the implications of the new graph-like genome assembly model for variant calling in the context of WGS. In particular, we investigate how stretches of a sequence that are largely but not entirely identical between the primary assembly and corresponding alternate loci affect variant calling in short-read (Illumina) WGS. The GRC Human Build 38 patch release 2 genome build (GRCh38.p2) contains a total of 178 genomic regions with one or more alternate loci; in total there are 261 alternate loci. In most cases (*n*=152), genomic regions had just one alternate locus, but five regions have five or more alternates: the CYP2D6 region with five alternate loci, REGION151 and the mucin region 2 with seven each, the MHC region with eight and the KIR gene family in the leukocyte receptor complex (LRC) region with 35. The regions range from 33,439 to 5,081,216 nt in length (mean 344,634 nt, median 169,569 nt), with most regions being between 100 and 200 kilobases (Fig. 1, Additional file 1: Supplemental Figures S1–S5, and Additional file 1: Supplemental Tables S1 and S2). The cumulative length of all of the 178 regions with alternate loci is 61,896,414 nt, which corresponds to about 2% of the primary assembly of the GRCh38 human genome (3,088,269,832 nt).

The 178 regions contain a total of 1120 unique genes, of which 797 are protein-coding genes. Moreover, 106 of these genes are associated with Mendelian diseases as listed in the Online Mendelian Inheritance in Man (OMIM) [22] resource. Additionally, 1145 of a total of 23,539 polymorphisms significantly associated with traits and common complex disease GWAS hits reported in the GWAS catalog [21] were located in the regions. These represented 353 diseases or traits. Altogether, 104 of the 178 regions contained at least one GWAS hit.

Therefore, incorrect calling of variants in these regions could have implications for the medical interpretation of genome data. The basic issue is that if a portion of an ALT-HAP is similar enough to the corresponding region of REF-HAP, then a read that originates from ALT-HAP may be falsely aligned to the REF-HAP. If the ALT-HAP sequence diverges from the REF-HAP sequence at a specific position, then this might lead to a spurious variant call at the corresponding position of REF-HAP, even though the read originates from ALT-HAP.

### Alignments of primary assembly regions with alternate loci

A comparison of the alignments between REF-HAP and corresponding ALT-HAP sequences reveals that they contain numerous stretches of alignable sequences that differ in numerous ways, including SNPs as well as small and large indels. Manual inspection suggested that the pairwise alignments between REF-HAP and ALT-HAP provided by GRC were not optimal in certain regions (Additional file 1: Supplemental Figures S6 and S7), and we, therefore, generated pairwise banded chain alignments between each of the 178 regions and the corresponding ALT-HAP sequences to refine the alignments (Additional file 1: Supplemental Figure S8 and “Methods”). This resulted in a total of 402 alignment blocks with a mean length of 248,928 nt (with respect to the REF-HAP sequence). There were 770,276 single- or multiple-nucleotide positions in the alignments that differed between REF-HAP and ALT-HAP, including 768,316 positions with differences less than 50 nt and 1960 structural differences of 50 nt and more (corresponding to 661,805 unique REF-HAP positions, since some REF-HAP regions can be aligned to multiple ALT-HAP loci). We will refer to these positions as ASDPs.

**Fig. 2:**
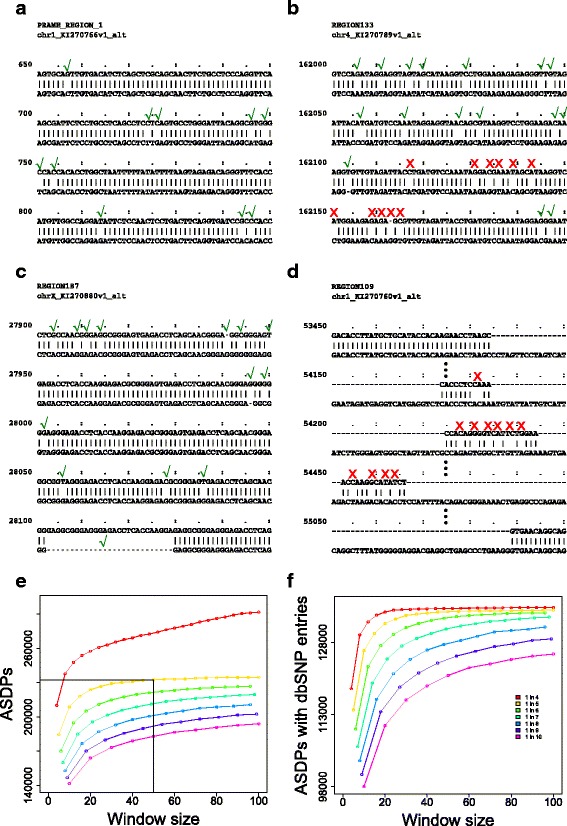
Frequency of ASDPs. Alignments contain stretches of sequences that are largely but not entirely identical between the primary assembly and an alternate locus, ranging from regions that are nearly identical to those with a substantial number of differences. ASDPs were defined to be positions of the alignment that differ between REF-HAP and ALT-HAP and are located in a sliding window in which at most 10 of 50 nucleotides are discrepant (*green check marks*). The *red crosses* show discrepancies that are excluded by this definition. In **a** and **c**, no ASDP was filtered out by the sliding window whereas in **b**, stretches of low sequence identity lead to the removal of several positions shown as *red crosses*. In **d**, large inserts in the ALT-HAP lead to a larger number of discrepant positions, which are discarded by the above criteria. **e** The effects of applying different thresholds of allowed discrepancies and window sizes to call ASDPs. The *dotted lines* mark the mismatch frequency (ten mismatches in 50 bases) used in this work. **f** Number of ASDPs that overlap with dbSNP variants according to the different thresholds. *ASDP* alignable scaffold-discrepant position

We reasoned that sequence reads corresponding to ALT-HAP loci are more likely to be aligned to the REF-HAP sequence if fewer differences between ALT-HAP and REF-HAP sequences exist. Examination of the alignments showed that some regions are identical or nearly so over up to several thousand nucleotides, while others display a greater number of discrepancies (Fig. 2 a–d). Each of these discrepancies potentially could lead to a variant call if an ALT-HAP read is misaligned to REF-HAP, but the actual likelihood of this occurring depends on many factors, including the overall degree of similarity of the REF-HAP and ALT-HAP positions in the corresponding region of the alignment. We, therefore, applied additional criteria for the goodness of the alignment in regions surrounding discrepant positions based on alignment windows that were allowed to contain up to a certain number of mismatches or gaps. We chose a threshold of 1 mismatch per 5 bases (1:5) since the total number of dbSNP entries that overlap with the discrepant position is nearly as high as with the 1:4 curve, which, however, is associated with a much higher overall number of variants (which we interpret as indicating a lower specificity). We chose a window size of 50 nt, since there was no substantial increase in the number of total discrepant positions or positions that overlap with dbSNP entries with larger window sizes. With these criteria, we identified a total of 232,333 alignment positions, which we will refer to as high-confidence ASDPs, and 187,080 of these ASDPs correspond to correspond to SNVs (80.5%), with the remainder representing indels ranging in size from 1 to 50 nt and about 4% block substitutions (Table 1). The total number of ASDPs is dependent on the window length and the number of allowed differences, but was relatively stable over a range of parameters (Fig. 2 e, f). In many cases, regions associated with multiple loci may have ASDPs originating from different ALT-HAP loci located at the same reference position. Such ASDPs may be identical or involve distinct nucleotide substitutions. All told, 137,156 unique REF-HAP positions, or ∼2.2 variations per kilobase of the REF-HAP sequence (61,896,414 bases in the 178 regions), are associated with an ASDP in one or more ALT-HAP loci.

**Table 1:** Distribution of ASDPs

ASDP category	Count	Percentage
SNV	187,080	80.5%
Deletion	15,955	6.9%
Deletion (1 nt)	6,368	2.7%
Deletion (2 nt)	2,413	1.0%
Deletion (3–50 nt)	7,174	3.1%
Insertion	15,286	6.6%
Insertion (1 nt)	6,423	2.8%
Insertion (2 nt)	2,224	1.0%
Insertion (3–50 nt)	6,639	2.9%
Block substitution	14,012	6.0%
Block substitution (2 nt)	11,659	5.0%
Block substitution (3 nt)	1,653	0.7%
Block substitution (4–50 nt)	700	0.3%

A total of 232,333 high-quality ASDPs were characterized by our algorithm of which 80.5% corresponded to SNVs when comparing the alternate locus with the corresponding primary assembly. About 7% each were deletions and insertions and 6% were block substitutions with equal numbers of nucleotides.

*ASDP* alignable scaffold-discrepant position, *SNV* single nucleotide variant

The acronym ASDP refers to a divergent position in the alignment between REF-HAP and ALT-HAP sequences, and not to a called variant. We will show that many variants called in WGS overlap with ASDPs, and we will refer to such variants as ASDP-associated variants. We restrict this analysis to the high-confidence ASDPs.

**Fig. 3:**
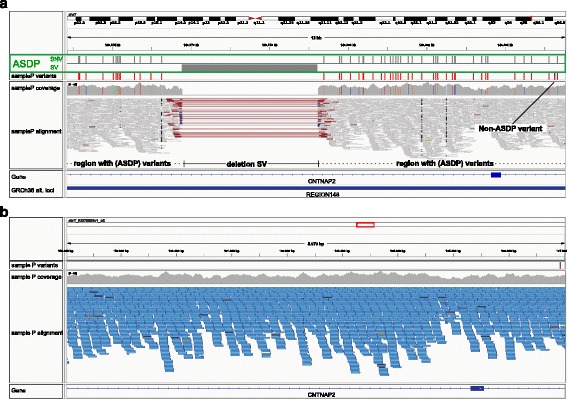
Region 148. IGV screenshots [50] are shown with variant calls for in-house sample *P*. **a** The presence of numerous ASDP-associated variants as well as a structural variant associated with the alternate locus KI270808.1 clearly suggest that the sample is homozygous for the KI270808.1 rather than for the REF-HAP sequence for region 148. Note that most of the variants that correspond to ASDPs are homozygous, suggesting that KI270808.1 is present in the homozygous state. An additional non-ASDP variant is present. Variants corresponding to 50 of the 52 ASDPs shown are listed in dbSNP. **b** The corresponding region on the alternate locus KI270808.1 was alignable well. Only the single non-ASDP-associated variant is called. IGV shows supplemental reads in *blue* (i.e., reads that map to the primary assembly as well as to an alternate locus). *ASDP* alignable scaffold-discrepant position, *SNV* single nucleotide variant, *SV* structural variant, *IGV* Integrative Genomics Viewer

In WGS data, the distribution of ASDP-associated in the 178 regions can be compared to a fingerprint that is indicative of the presence of one of the ALT-HAP sequences, the REF-HAP sequence, or their heterozygous combination. That is, ASDPs are associated with characteristic patterns of variant calls against the REF-HAP sequence. Figure 3 shows an example of how ASDPs affect variant calling in an in-house genome. In Fig. 3 a, multiple homozygous variants called against region 148 on chromosome 7q correspond to ASDPs with a single heterozygous non-ASDP-associated variant. Figure 3 b shows the corresponding sub-region of alternate locus KI270808.1, which is assigned to region 148. Only the single (heterozygous) non-ASDP variant is called. Therefore, it is a plausible inference that the sequenced proband is homozygous for the locus KI270808. Furthermore, the variants called against the REF-HAP sequence in this region (region 148) are likely to be spurious in the sense that the sequenced individual does not have the canonical chromosome 7p sequence (REF-HAP) in this region, but instead has KI270808.1 (ALT-HAP). Assuming the variant call against KI270808.1 is accurate, then the individual has only this single variant against KI270808.1.

### Postprocessing VCF files to infer ASDPs

Here, we present a heuristic algorithm that infers the presence of ALT-HAP sequences in WGS data based on the distribution of ASDP-associated variants in the VCF file [23]. We chose to use VCF files as input to our algorithm, since VCF files with variants called from exome or genome sequencing data are commonly used as a standard format for interpretive software such as Exomiser [24–26]. We, therefore, developed an algorithm to postprocess VCF files from WGS to identify REF-HAP and ALT-HAP genotypes and to flag ASDP-associated variant calls. The algorithm takes as input a VCF file produced from a variant caller such as the GATK haplotype caller [14] or FreeBayes [13] that is applied to an alignment produced by BWA-MEM [15] (see “Methods”). We call our algorithm ASDPex, because it is designed to extract ASDP-associated variants from VCF files. There may be analysis goals for which it would be appropriate to remove ASDP-associated variants from further analysis. For instance, one might want to remove the 52 variants called against region 148 (Fig. 3 a) and retain only the single non-ASDP-associated variant called against KI270808.1.

**Fig. 4:**
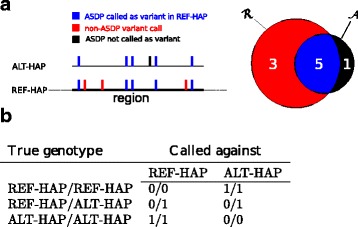
Overview of the ASDPex algorithm. **a** ASDPex compares the set of all variants called against REF-HAP $\mathcal {R}$ with the set of ASDPs associated with ALT-HAP $\mathcal {A}$. In this example, $\left | \mathcal {A}\right |$ (the number of ASDPs associated with ALT-HAP) is 6, and $\left |\mathcal {R}\right |$ (the total number of variants called against REF-HAP) is 8. ASDPex defines the set of residual variants as the symmetric set difference between $\mathcal {R}$ and $\mathcal {A}$, i.e., $\text {\texttt {RV}}=\mathcal {R} \triangle \mathcal {A}$. Therefore, |RV|=4, and because $\left |\text {\texttt {RV}}\right | < \left |\mathcal {R}\right |$, our algorithm infers that ALT-HAP is present. **b** The pattern of variant calls obtained for ASDPs differs according to whether the sequenced proband is homozygous for one of the two alternate loci or is heterozygous. Our algorithm exploits this pattern across the entire length of the alternate locus to infer the most likely genotype. *ASDP* alignable scaffold-discrepant position

ASDPex is a heuristic algorithm that compares the distribution of ASDPs and other variants in the REF-HAP and ALT-HAP sequences to infer the most likely genotype of each region (i.e., homozygous REF-HAP, homozygous ALT-HAP for one of the alternate loci, or heterozygous). ASDPex scans each of the 178 regions (i.e., REF-HAP) in turn and compares all of the associated alternate loci (ALT-HAP). ASDPex considers two sets. Let $\mathcal {R}$ be the set of all variants called for the REF-HAP. Note that these variants may include both ASDP-associated variants as well as additional variants. Let $\mathcal {A}$ be the set of all ASDPs for the corresponding REF-HAP to ALT-HAP alignment (Fig. 4). If the sample being sequenced does not contain the ALT-HAP, then we do not expect the variants called against REF-HAP to contain many ASDP-associated variants, and instead interpret all the variants in the set $\mathcal {R}$ as true positives. On the other hand, if the sample being sequenced does contain the ALT-HAP, then we expect that many of the variants called against REF-HAP to be ASDP-associated variants. Our algorithm then interprets the residual variants (RV), comprising all non-ASDP-associated variants called against REF-HAP as well as all ASDPs associated with the ALT-HAP that are *not* called against the REF-HAP, as true positives. The set RV can be calculated as the symmetric difference of the sets $\mathcal {R}$ and $\mathcal {A}$. Our algorithm finally infers the presence of ALT-HAP if this would reduce the total number of called variants, i.e., if $\left | \text {\texttt {RV}}\right | < \left |\mathcal {R}\right |$ (see Fig. 4 and “Methods”).

If most of the ASDP-associated variants are called as homozygous against the REF-HAP (by default 90%), our heuristic assumes the ALT-HAP is present in a homozygous state, and otherwise it is in a heterozygous state. If a REF-HAP region is associated with more than one ALT-HAP, then the ALT-HAP associated with the greatest reduction in ASDP-associated variants is chosen. Finally, ASDPex outputs a VCF file in which regions and variants are correspondingly marked such that they can be filtered in downstream analysis if desired (see “Methods” and Additional file 1: Supplemental Figure S11 for details).

The genotype shown in Fig. 3 was inferred to be homozygous ALT-HAP by ASDPex. Additional file 1: Supplemental Figures S12–S22 show additional examples in which ASDPex inferred an alternate locus to be present in the homozygous or heterozygous state or inferred homozygous REF-HAP.

### Population frequencies of alternate loci

The ASDPex algorithm infers whether the canonical chromosome sequence or an ALT-HAP sequence is most likely given the observed pattern of variants and ASDPs.

To investigate whether the alternate loci show population biases, we used ASDPex to analyze 30 WGS samples each from four populations from the 1000 Genomes Project [18]. We observed that several alternate loci showed a highly specific population bias (Table 2). The Peruvian population displayed the highest number of population-specific alternate loci. The lowest mean count of alternate loci was found in European and African samples, possibly because these populations have been extensively studied and are well represented in the current genome assembly (Additional file 1: Supplemental Figure S23).

**Table 2:** Population-specific alternate loci

Alternate locus	FIN	LWK	CHB	PEL
chr4_KI270787v1_alt			$\checkmark $	$\checkmark $
chr5_GL383531v1_alt	$\checkmark $			
chr5_GL949742v1_alt			$\checkmark $	
chr6_GL383533v1_alt				$\checkmark $*
chr6_KI270801v1_alt		$\checkmark $	$\checkmark $	$\checkmark $*
chr9_GL383542v1_alt		$\checkmark $		
chr11_JH159136v1_alt		$\checkmark $		$\checkmark $*
chr13_KI270839v1_alt	$\checkmark $		$\checkmark $	$\checkmark $*
chr14_KI270844v1_alt			$\checkmark $	$\checkmark $
chr15_GL383555v2_alt	$\checkmark $*		$\checkmark $	$\checkmark $*
chr18_GL383570v1_alt			$\checkmark $	

Shown are all the alternate loci that were inferred to be present in at least 90% of the individuals of a population. Alternate loci present in all investigated individuals of the population are marked with an asterisk (*)

*CHB* Asian, Han Chinese in Beijing, China, *FIN* European, Finnish in Finland, *LWK* African, Luhya in Webuye, Kenya, *PEL* South Americans, Peruvians from Lima, Peru

### ASDPs in dbSNP

We then investigated SNPs and other polymorphisms that map to the 178 regions in dbSNP [10], which contains 35,171,619 common SNP entries. Of these, 826,612 were located within the 178 REF-HAP regions. A total of 75,138 of these overlap with an ASDP, including 71,653 unique REF-HAP variants, which is about 32.3*%* of all ASDPs (Additional file 1: Supplemental Table S3).

The GWAS Catalog [21] contains 18,130 unique SNPs (GWAS hits) with chromosomal coordinates which are significantly associated with a disease or trait at a *p* value of less than 10^−5^. Altogether, 791 GWAS hits are within the 178 regions associated with alternate locus regions, and 437 GWAS hits were found to overlap with ASDPs, 360 of which are located within the MHC region (Additional file 1: Supplemental Table S4).

### Variant calling on 121 in-house genomes: GRCh37 vs. GRCh38

In addition to the increased numbers of alternate loci in the GRCh38 build as compared to the GRCh37 build, there are many other differences, including numerous corrections and the addition of sequences to close many gaps in the GRCh37 build. We investigated the performance of variant calling on 121 in-house genomes that were processed using BWA-MEM [15] for alignment and FreeBayes [13] for variant calling. Except for the reference genome sequence used, all processing steps were identical (see “Methods” for details). We restricted the analysis to the chromosomes of the primary assembly except as noted below. In both cases, the overall quality was good, with 99.8% of the reads being mapped. Reads that can be mapped equally well to two or more positions in the target are referred to as supplementary mapped reads. As expected, a major difference in the alignment was the presence of a mean of nearly 100 times more supplementary reads for the GRCh38 alignment than for the GRCh37 alignment.

**Fig. 5:**
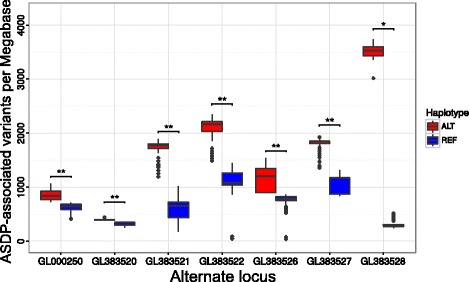
Distribution of ASDP-associated variants called against the primary assembly. A significantly and substantially higher number of ASDP-associated variants are called against the primary assembly according to whether the region is inferred to be REF-HAP or ALT-HAP by the ASDPex algorithm. The data appear to fall into two well-separated clusters. The figure shows the counts of Ref/Alt ASDP-associated variants per megabase for seven selected regions for the 121 in-house genomes. * *p*<1×10^−8^; ** *p*<1×10^−10^ (Mann–Whitney test). *ASDP* alignable scaffold-discrepant position

The number of variant calls between the genome releases and the mean Phred scores were comparable (Table 3). Variants that correspond to ASDPs can be found in samples with regions that are inferred to correspond to the primary assembly. However, the density of ASDPs called against regions of the primary assembly is substantially and significantly different according to whether the region was inferred to correspond to the primary assembly or an alternate locus (Fig. 5).

**Table 3:** Variant statistics for both genome builds

	GRCh37				GRCh38			
Chromosome	All	Common	Rare	Phred	All	Common	Rare	Phred
1	344158	299500	44659	503.42	359530	291704	67825	473.83
2	354113	247585	106528	506.54	361469	243400	118069	492.91
3	295447	268021	27426	503.76	301985	263993	37993	492.99
4	319988	290080	29908	515.41	324134	285405	38729	507.33
5	266077	235462	30615	498.58	272482	231747	40734	485.57
6	280789	252705	28084	495.02	279132	246545	32588	487.11
7	249980	220543	29437	488.95	257917	216669	41248	475.69
8	229332	204823	24509	499.70	229541	200845	28696	490.15
9	192615	162202	30413	475.09	200034	159119	40916	466.46
10	217957	194385	23572	508.74	229352	190658	38694	494.18
11	219134	197412	21722	522.84	228324	194132	34192	498.04
12	205085	184477	20608	502.78	212789	175990	36799	483.94
13	166128	151271	14856	530.31	180521	148870	31651	494.65
14	141971	124790	17181	503.15	140443	122524	17919	495.75
15	130324	112085	18239	505.95	131389	109741	21648	493.46
16	134293	116224	18069	487.40	136799	113589	23210	473.36
17	118096	102300	15796	479.64	130637	99074	31563	452.99
18	124509	111958	12552	516.80	132628	110349	22279	485.89
19	98104	84416	13688	456.51	99625	82875	16750	455.35
20	90490	79709	10781	486.09	112562	78562	33999	475.40
21	69511	55211	14300	525.23	73027	53052	19975	513.27
22	59660	50242	9418	455.99	71112	48961	22151	445.27
Total	4307761				4465432			

The mean counts of autosomal variants and the median Phred scores per chromosome are shown for GRCh37 and GRCh38.

Columns: All: all detected variants; Common: listed in dbSNP common_all_*; Rare: variants that are not common.

The mean variant counts for chromosome X were 127,914 (GRCh37) and 132,177 (GRCh38). For chromosome Y, the mean counts could not be estimated since gender information was not available for all of the 121 in-house genomes. Both genome releases include the identical mitochondrial reference (*NC_012920.1*) with 27 variants

The transition/transversion (Ts/Tv) ratio, a commonly used quality control parameter for checking overall SNP quality, is often found to be around 2.0 across the whole genome [27, 28]. We calculated the Ts/Tv ratio for both genome releases and found it to be 2.03 for GRCh37 and 1.99 for GRCh38. This difference is related to the substantially lower Ts/Tv ratio associated with rare variants (Additional file 1: Supplemental Figure S24).

There was a mean of 24908±380 variants overlapping protein-coding regions (CDS) in the 121 in-house genomes when analyzed with the GRCh37 genome. Analysis with the GRCh38 genome revealed slightly more CDS variants, 26499±421.

Finally, we investigated the effects of ASDPs on variant calling in the GRCh38 build. For this analysis, we included only regions for which the GRCh38 build had at least one alternate locus. We did not include region 116, because no alignable sequence in the GRCh37 build was identified. We applied ASDPex to each of the VCF files resulting from variant calling in the 121 in-house genomes. Of the 178 regions, 51.8±3.8 (range 38–60) were found to correspond best to an alternate locus rather than the primary assembly sequence. A mean of 7863 variant calls were found to be ASDP-associated per genome, or 6.51*%* of all variants located in the 178 regions (Table 4). Although many of these ASDP-associated variants are listed in dbSNP, about 13% of all ASDP-associated variants called per genome were not (Additional file 1: Supplemental Figure S25). A small number of the ASDP-associated variants that are not listed in dbSNP were predicted to have high impact (Additional file 1: Supplemental Table S5).

**Table 4:** Reduction in called variants by ASDPex

Variant calling pipeline	Total variants	Variants per Mb
GRCh37 canonical	114,023 ± 4,983	2198.3 ± 207.6
GRCh38 canonical	120,807 ± 4,069	1975.2 ± 66.5

The variant counts are shown for 121 in-house whole-genome sequencing samples in the ALT-LOCI-containing regions. For GRCh37, a liftover of the regions was performed and region 116 was removed from both datasets, since no alignable region(s) are present in GRCh37. Since the size of the regions is different in GRCh37 and GRCh38, average variant counts per megabase (Mb) are also shown. On average, there was a reduction of 7863 ± 2675 (6.5%) variants called using ASDPex in the ALT-LOCI-containing regions, corresponding to a reduction from 1975.2 ± 66.5 to 1846.7 ± 71.6 variants per Mb

## Discussion

Variant calling is required for medical interpretation, which focuses on sequences that diverge from normal. As WGS develops into a tool for clinical diagnostics [29–31], there is a pressing need to improve the technical accuracy of sequencing methods and analysis pipelines [32]. Numerous challenges need to be addressed to achieve this goal, including the low concordance rate of alignment and variant-calling pipelines [32, 33]. In this work, we have characterized ASDPs, which correspond to differences in alignments between sequences that are largely but not entirely identical between the primary assembly and an alternate locus. ASDPs are associated with characteristic patterns of variant calls against the primary assembly and corresponding alternate loci. The ASDP-associated variants identified by our analysis can be commonly found in WGS data (Fig. 3, Additional file 1: Supplemental Figures S12–S22, and Table 4).

### Do ASDPs lead to spurious variant calls?

Variant calling is always contextual, and whether something is a variant will depend on the reference sequence used. For instance, over 10,000 sites had a base change between GRCh37 and GRCh38, so some sites that were variant in GRCh37 will not be called as variant in GRCh38. For the most part, these variants are simply errors in GRCh37, and have been corrected in GRCh38 (including some positions such as chr15:48807637C).^1^ The ASDP-associated variants characterized in this work are not false positive in this sense. Instead, the distribution of ASDP-associated variants in the 178 regions can be compared to a fingerprint that is indicative of the presence of one of the ALT-HAP sequences, the REF-HAP sequence, or their heterozygous combination. The distribution of ASDP-associated variants can be used to infer that variant calls against a structural variant region of the primary assembly of the GRCh38 assembly are spurious in the sense that the sample contains an alternate locus at that segment of the genome. For instance, in the example shown in Fig. 3, the variants called against the chromosome sequence of the primary assembly can be considered to be spurious, since it is much more likely that the sequenced individual is homozygous for the alternate locus KI270808.1.

**Fig. 6:**
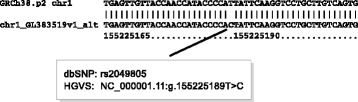
rs2049805. The GWAS hit rs2049805 corresponds to an ASDP defined by an alignment between chromosome 1 of the primary assembly (region MTX1) and the alternate locus GL383519.1, which is identical over a stretch of 49 nucleotides except for the middle position. rs2049805 is significantly associated with blood urea nitrogen levels in east Asian populations [51]. *ASDP* alignable scaffold-discrepant position

Accurate calling of variants in these positions will depend on there being enough sequence context to locate unambiguously the data upon which the variant call is made. This may not be possible for technologies such as Affymetrix SNP-Chips [35, 36] (Fig. 6), which by design interrogate a stretch of 49 nt. Given the overall similarity of the REF-HAP and ALT-HAP sequences in the vicinity of many of the ASDPs that overlap with GWAS hits, it is conceivable that current SNP measurement technologies such as Affymetrix are identifying sequences on ALT-HAPs and not (or not just) variants located in the REF-HAP sequence. Altogether, 139 of the 437 GWAS hits that overlap with ASDPs are located in regions of the alignment that are identical in a region of 25 nt up- and downstream with the exception of the ASDP itself, raising the question of whether these GWAS hits are actually tagging the presence of an ALT-HAP rather than a polymorphism linked to the reported location in the golden-path chromosome (Fig. 6). The great majority of GWAS hits are not themselves disease-causative but rather tag susceptibility regions (haplotypes that contain one or more deleterious variants). If, in fact, the alternate loci are associated with the trait, rather than a particular haplotype of the corresponding region of the primary assembly, then searches for the causative variants associated with GWAS hits need to take the sequences of the alternate loci into account.

The finding that a single variant colocalizes with an ASDP is not in itself indicative that the variant is spurious or a false positive. In fact, our results suggest that the primary assembly may contain polymorphisms whose alternate alleles correspond to the sequence in an alternate locus (because we identified ASDP-associated variants in sequences inferred to be a primary assembly; see Fig. 6). A limitation of the current study is that we have not attempted to analyze the frequency of recombination between REF-HAP and ALT-HAP in the population. Recombination between the different loci at a structurally variable genomic region may be one reason why ASDP-associated variants can be found on the background of haplotypes inferred to be REF-HAP. Our algorithm is based on the simplifying assumption that the alternate loci represent complete haplotype blocks. However, linkage disequilibrium blocks for Europeans are ∼60 kb on average (and less for African populations) [37]. Therefore, it is possible that the alternate loci are not always a valid haplotype observed within the population. The frequency of recombination events between regions of the primary assembly and the corresponding alternate loci has yet to be studied in detail. Another limitation of our analysis is that our definition of ASDPs depends on the accuracy of the alignment and the parameters used to define ASDPs. Although the majority of ASDPs identified by our algorithm were SNVs or small indels (Table 1), it is possible that more sophisticated methods of identifying complex rearrangements [38] between REF-HAP and ALT-HAP sequences may make it possible to identify corresponding ASDP-associated variants.

### Challenges and opportunities for variant calling with the GRCh38 genome assembly model

Our work has illustrated some of the difficulties that ensue when calling variants with the GRCh38 genome assembly model. To address these challenges, the community will need to decide upon the desired output from variant callers. The current strategy recommended by GATK is to use BWA-MEM to align reads to both the primary assembly and the alternate loci followed by variant calling for the primary assembly and the alternate loci separately using GATK. Current pipelines do not attempt to disambiguate variant calls in regions of the genome associated with alternate loci. We have presented a downstream tool, ASDPex, that can process such output with the ambiguities that result from this approach and that would allow processing of ASDP-associated variants. It is also possible to imagine that variant calling tools be required to infer which haplotypes (REF-HAP or ALT-HAP) are present before calling variants; this process could take advantage not only of knowledge about structural variation in the human genome, as ASDPex does, but also could use information in the alignment (BAM) file itself to perform variant calling simultaneously with an analysis of reads with supplemental mappings. The community would need to agree upon the best way of representing these results in VCF format (see Additional file 1: Supplemental Figure S11 for the conventions used by ASDPex).

There are several limitations to the current VCF file format that make it difficult to represent data fully using the GRCh38 assembly. The SAM format can represent supplementary alignments representing reads that map to an alternate locus in addition to the primary assembly (Additional file 1: Supplemental Figure S26). An analogous extension to the VCF format does not exist. The current format does not represent the relationship between alternate alleles and their chromosome locations (i.e., maintaining the allelic relationships of the alternate sequences). While this is a valid requirement within a specific assembly unit, it needs to be relaxed when describing data on the full assembly as a feature can validly be on the primary assembly as well as an alternate locus.

The VCF format can represent hemizygous variants on the X chromosome in males as 1, although in practice such variants are often called homozygous (1/1) by variant callers that are not aware of the sex of the proband. If a sample was inferred to be heterozygous for REF-HAP and ALT-HAP, then one potential way of representing variants called against both haplotypes would be with the 1 notation.

It is possible that human genome sequencing will move towards de novo assembly of genomes as technologies and bioinformatic analysis strategies mature [39], a step that would likely require substantial evolution of current file formats and analysis pipelines to be applied in diagnostic settings.

Despite the fact that the GRCh38 genome build has been available for over 18 months, progress in using GRCh38 in medical interpretation pipelines has been slow. We suggest that this is a chicken and egg problem. If we want variant callers to be able to use the alternate loci, we need to be able to express the variants in VCF, which does not work well with the current specification. Ultimately, new models of representing variation in the genome may be required [40].

## Conclusions

The work presented here is an exploration of the implications of the new genome build for variant calling in WGS analysis. We have presented a simple heuristic algorithm designed to search for characteristic distributions of ASDP-associated variants in VCF files that allow the presence of alternate loci to be inferred. We have argued that variants called against the primary assembly may be spurious in some such cases.

Accurate assembly of genomes is essential for the understanding of genetic variation [39]. The GRCh38 genome assembly was a major step towards developing a model that can adequately represent the structural variation in the human population. However, it is likely that there is a substantially higher number of regions in the human genome that exhibit a degree of structural variability that cannot be adequately represented by a linear genome model. Current work with long-read technologies and algorithmic advances are resolving an increasing number of genomic regions, which may be incorporated into future assemblies of the human genome [39, 41–47]. As our knowledge of the human genome and its variation in population increases, it seems likely that more sophisticated graph-based representations of the genome will become useful [40, 48].

Our results suggest that it could be useful to develop algorithms that infer the most likely diplotype of each of the 178 regions associated with alternate loci and instead realign the original reads with the alternative haplotype with the primary where indicated, and present the variant calls accordingly. While this procedure ultimately may be replaced by full de novo diagnostic genome assembly or variant calling strategies related to graph-based representations, this procedure could be done with the tools available today.

The pilot project presented in this works suggests the great potential of fully incorporating the resources of graph-like genome assemblies into variant calling, but also underscore the importance of developing computational resources that will allow full reconstruction of the genotype in personal genomes.

## Endnote

^1^ If the wild-type RefSeq for the *FBN1* gene, NM_000138, is compared to the genomic sequence, a variant c.1415G >A;p.Tyr472Cys would be called that is predicted to be pathogenic [34]. The genomic base has been corrected to a T in GRCh38.

## Additional file

Additional file 1 Online supplemental material. A file with 26 figures, five tables, and two algorithms. (PDF 24300 kb)

## Abbreviations

ASDP: Alignable scaffold-discrepant position
DNA: Deoxyribonucleic acid
GFF: General feature format
GRC: Genome Reference Consortium
GWAS: Genome-wide association study
indel: Insertion or deletion
LRC: Leukocyte receptor complex
MHC: Major histocompatibility complex
NCBI: National Center for Biotechnology Information
nt: nucleotides
OMIM: Online Mendelian Inheritance in Man
SAM: Sequence alignment/map
SNP: Single nucleotide polymorphism
SNV: Single nucleotide variant
SV: Structural variant
VCF: Variant calling format
WGS: Whole-genome sequencing
