# Supplementary material for: Alternate-locus aware variant calling in whole genome sequencing
Source: Genome Med. 2016 Dec 13;8:130. doi: 10.1186/s13073-016-0383-z (PMC5155401; doi:10.1186/s13073-016-0383-z)
Supplement: Additional file 1 — Online supplemental material. A file with 26 figures, five tables, and two algorithms. (PDF 24300 kb) [file 13073_2016_383_MOESM1_ESM.pdf]

Alternate-Locus Aware Variant Calling in Whole Genome Sequencing

Online Supplement

November 21, 2016

Contents

	Page
**Figure S1** ALC Region	2
**Figure S2** REGION151	3
**Figure S3** MHC region	4
**Figure S4** REGION14	5
**Figure S5** REGION142	6
**Figure S6** NCBI Alignment for REGION155	7
**Figure S7** Corrected Alignment for REGION155	8
**Figure S8** Banded alignment	9
**Figure S9** Anchors for alignments	10
**Figure S10** First-pass screening for candidate ASDPs	11
**Figure S11** ASDPex VCF file extensions and modifications	12
**Figure S12** REGION176 vs. KI270859.1	13
**Figure S13** REGION165 vs. KI270839.1	14
**Figure S14** REGION123 vs. KI270780.1	15
**Figure S15** ZNF66 vs. GL383573.1	16
**Figure S16** REGION42 vs. GL383579.2	17
**Figure S17** REGION112 vs. KI270759.1	18
**Figure S18** REGION112 vs. KI270759.1	19
**Figure S19** REGION108 vs. KI270762.1	20
**Figure S20** SERPIN_REGION_1 vs. KI270845.1	21
**Figure S21** ABR vs. KI270910.1	22
**Figure S22** REGION23 vs. GL383552.1	23
**Figure S23** Number of annotated alternate loci per population	24
**Figure S24** Ts/Tv ratio for both genome builds	25
**Figure S25** ASDP-associated variants in 121 in-house genomes	26
**Figure S26** SAM format for read with supplementary alignment	27
**Table S1** Fields of "alt_scaffold_placement.txt"	28
**Table S2** Size distribution of the alternate scaffolds of GRCh38.p2	29
**Table S3** Sequence ontology categories of ASDPs	30
**Table S4** GWAS Hits that Overlap with ASDPs	31
**Table S5** High-impact Alignable Scaffold-Discrepant Positions (ASDPs) not listed in dbSNP	39
**Algorithm S1** Determine candidate seed	40
**Algorithm S2** ASDPex	41

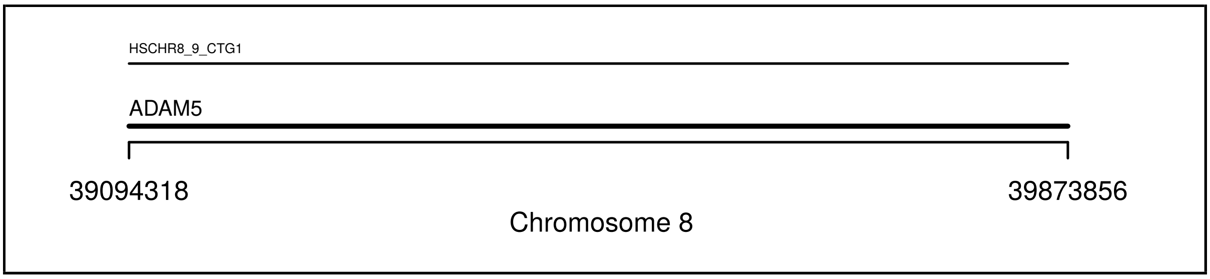

Field	Value
`alt_asm_name`	`ALT_REF_LOCI_1`
`prim_asm_name`	`Primary Assembly`
`alt_scaf_name`	`HSCHR8_9_CTG1`
`alt_scaf_acc`	`NT_187577.1`
`parent_type`	CHROMOSOME
`parent_name`	8
`parent_acc`	`NC_000008.11`
`region_name`	ADAM5
`ori`	+
`alt_scaf_start`	1
`alt_scaf_stop`	624492
`parent_start`	39094318
`parent_stop`	39873856
`alt_start_tail`	0
`alt_stop_tail`	0

Figure S1: ALC Region. A single alternate locus defines start and stop positions of the ALC region. In this example, the region called ADAM5 is located on chromosome 8 (`NC_000008.11`) at positions 39,094,318–39,873,856 (corresponding to 779,539 nucleotides). The alternate scaffold comprises 624,492 nucleotides. The table shows the values of the `alt_scaffold_placement.txt` file that describe the alternate scaffold. See **Table S1** for an explanation of the fields.

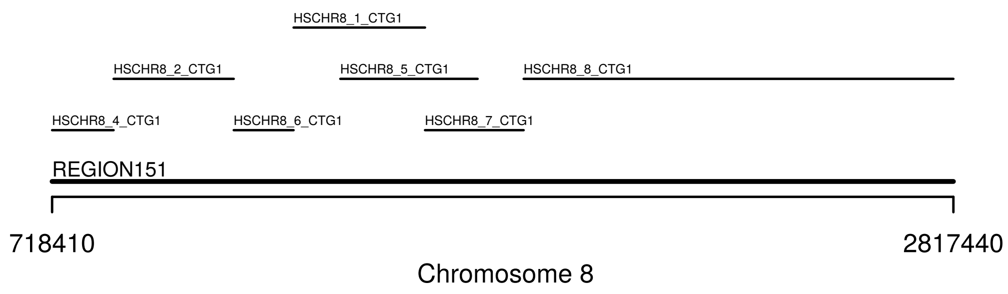

Field	Value
alt_asm_name	ALT_REF_LOCI_1
prim_asm_name	Primary Assembly
alt_scaf_name	HSCHR8_4_CTG1
alt_scaf_acc	NT_187572.1
parent_type	CHROMOSOME
parent_name	8
parent_acc	NC_000008.11
region_name	REGION151
ori	+
alt_scaf_start	1
alt_scaf_stop	145606
parent_start	718410
parent_stop	861641
alt_start_tail	0
alt_stop_tail	0

Figure S2: REGION151. REGION151 is located on chromosome 11 (NC_000008.11) at positions 718,410–2,817,440 (corresponding to 2,099,031 nucleotides). Multiple alternate scaffolds can be contained in this region. The table shows REGION151, which corresponds to positions 718,410–861,641 (143,232 nucleotides) on chromosome 8, and comprises a total of 145,606 nucleotides. Each of the seven alternative scaffolds was aligned separately, for a total of seven different alignments. The regions not covered by the alignment are assumed to be identical with the reference sequence. See **Table S1** for an explanation of the fields.

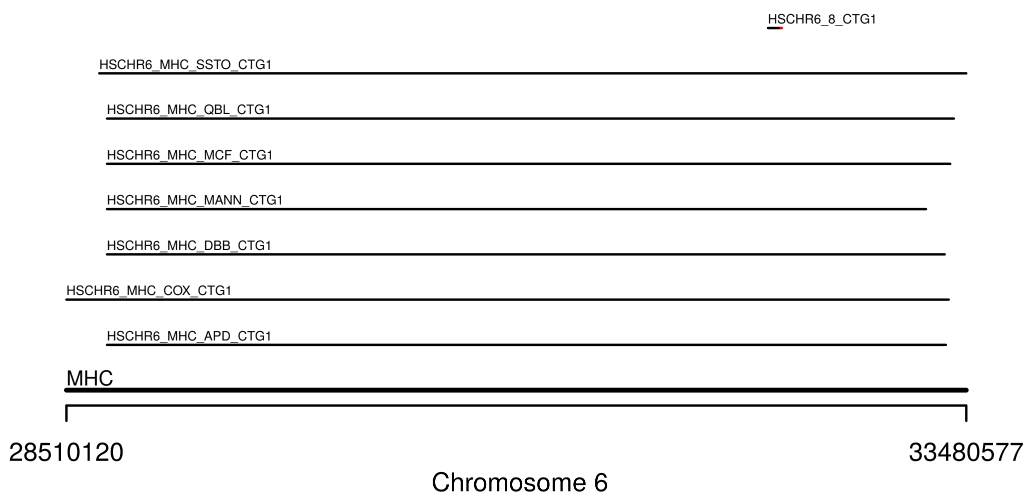

Field	Value
alt_asm_name	ALT_REF_LOCI_1
prim_asm_name	Primary Assembly
alt_scaf_name	HSCHR6_MHC_APD_CTG1
alt_scaf_acc	NT_167244.2
parent_type	CHROMOSOME
parent_name	6
parent_acc	NC_000006.12
region_name	MHC
ori	+
alt_scaf_start	1
alt_scaf_stop	4672374
parent_start	28734408
parent_stop	33367716
alt_start_tail	0
alt_stop_tail	0

Figure S3: MHC. The MHC region is located on chromosome 6 (NC_000006.12) at positions 28,510,120–33,480,577 (corresponding to 4,970,458 nucleotides). The table shows HSCHR6_MHC_APD_CTG1, which comprises 4,672,374 nucleotides. See **Table S1** for an explanation of the fields.

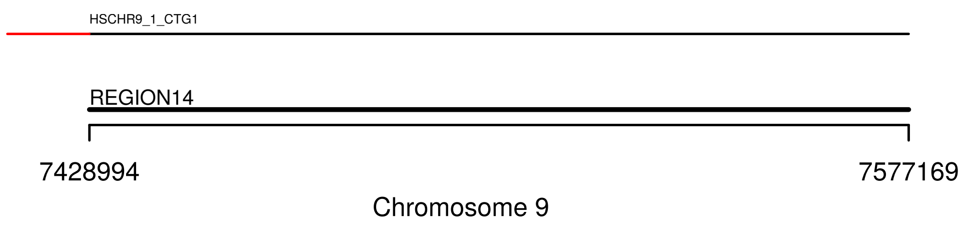

Field	Value
alt_asm_name	ALT_REF_LOCI_1
prim_asm_name	Primary Assembly
alt_scaf_name	HSCHR9_1_CTG1
alt_scaf_acc	NW_003315928.1
parent_type	CHROMOSOME
parent_name	9
parent_acc	NC_000009.12
region_name	REGION14
ori	+
alt_scaf_start	14845
alt_scaf_stop	162988
parent_start	7428994
parent_stop	7577169
alt_start_tail	14844
alt_stop_tail	0

Figure S4: REGION14. REGION14 is located on chromosome 9 (NC_000009.12) at positions 7,428,994–7,577,169 (corresponding to 148,176 nucleotides). The segment shown in red is an insertion. See **Table S1** for an explanation of the fields.

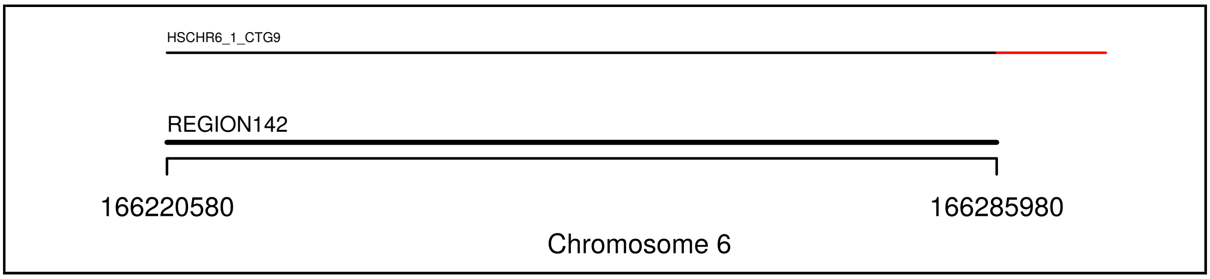

Field	Value
alt_asm_name	ALT_REF_LOCI_1
prim_asm_name	Primary Assembly
alt_scaf_name	HSCHR6_1_CTG9
alt_scaf_acc	NT_187557.1
parent_type	CHROMOSOME
parent_name	6
parent_acc	NC_000006.12
region_name	REGION142
ori	+
alt_scaf_start	1
alt_scaf_stop	66404
parent_start	166220580
parent_stop	166285980
alt_start_tail	0
alt_stop_tail	8601

Figure S5: REGION142. REGION142 is located on chromosome 6 (NC_000006.12) at positions 166,220,580–166,285,980 (corresponding to 65,401 nucleotides). The segment shown in red is an insertion. See **Table S1** for an explanation of the fields.

```
12600     .    :    .    :    .    :    .    :    .    :
      TAGTCCATGTGCTGTGGTTGGGGCTGGCATTGCTGGTGTTGGCATTGGTG
      ||||||||||||||||||||||||||||||||||||||||||||||||||
      TAGTCCATGTGCTGTGGTTGGGGCTGGCATTGCTGGTGTTGGCATTGGTG

12650     .    :    .    :    .    :    .    :    .    :
      TTGTTGGGGTTGGGGTTGGTGGTGGTGGTGGTGGTGTGGTGTTGTTGGTG
      ||||||||||||||||||||||||||||||||||||||||||||||||||
      TTGTTGGGGTTGGGGTTGGTGGTGGTGGTGGTGGTGTGGTGTTGTTGGTG

12700     .    :    .    :    .    :    .    :    .    :
      TTAGTGTTGGTGTTCTGATGTGGTGTTGGTGGTGTTGGTGTTGGTGGTGT
      ||||||||||||||||||||||||||||||||||||||||||||||||||
      TTAGTGTTGGTGTTCTGATGTGGTGTTGGTGGTGTTGGTGTTGGTGGTGT

12750     .    :    .    :    .    :    .    :    .    :
      TGTTGGTGTGGTGGTGTTGGTGTGGTGTTGTTGGTGTTGGTGTTGGTGGT
      ||||||||||||||||||||||||||||||||||||||||||||||||||
      TGTTGGTGTGGTGGTGTTGGTGTGGTGTTGTTGGTGTTGGTGTTGGTGGT

12800     .    :    .    :    .    :    .    :    .    :
      GTTGGTGTTGGTGTGGTGTTGTTGGTGTTGGTGTGGTGTGATGATGGTGG
      |||||||||||||||||||||||||||||||||||||||   | || |||
      GTTGGTGTTGGTGTGGTGTTGTTGGTGTTGGTGTGGTGTT--GGTGTTGG

12850     .    :    .    :    .    :    .    :    .    :
      TGGTATTGTGGTAATAGGATGGTGGTGGTAGTATGGTGGTGGT-------
      |||| |||  ||  | || || ||||| |  |  |||| ||||
      TGGTGTTGGTGTTGTTGG-TGCTGGTGTTGTT--GGTGTTGGTGTGGTGT

     ...

16100     .    :    .    :    .    :    .    :    .    :
      ----GGTGGTAACAGTAGGCATGGTGGTGATTACTATATAACGATGATAA
          ||||||||||||||||||||||||||||||||||||||||||||||
      TGGTGGTGGTAACAGTAGGCATGGTGGTGATTACTATATAACGATGATAA

16150     .    :    .    :    .    :    .    :    .    :
      CGTTCTTGTGGAGGTGACTGTGTCGATGACACACTTGTGTGTCTGATGCC
      ||||||||||||||||||||||||||||||||||||||||||||||||||
      CGTTCTTGTGGAGGTGACTGTGTCGATGACACACTTGTGTGTCTGATGCC

16200     .    :    .    :    .    :    .    :    .    :
      AAGTGCTCCCTGGACTTCTGTTTGCTCCTGGGCTCTTTTCCATCCCAGCT
      ||||||||||||||||||||||||||||||||||||||||||||||||||
      AAGTGCTCCCTGGACTTCTGTTTGCTCCTGGGCTCTTTTCCATCCCAGCT

16250     .    :    .    :    .    :    .    :    .    :
      CTTCACCAGAGGGAGTTTTCATTTCCTGAGAGGGCGGCTGCTCTGATTGC
      ||||||||||||||||||||||||||||||||||||||||||||||||||
      CTTCACCAGAGGGAGTTTTCATTTCCTGAGAGGGCGGCTGCTCTGATTGC
```

Figure S6: This example shows the alignment implied by the NCBI GFF file for a portion of the alignment of `chr8_KI270820v1_alt` (bottom) against `REGION155` (top). Manual inspection suggests that this alignment is not optimal after position 12,839 (see also **Figure S7**).

```
12650      .    :    .    :    .    :    .    :    .    :
       TTGTTGGGGTTGGGGTTGGTGGTGGTGGTGGTGGTGTGGTGTTGTTGGTG
       ||||||||||||||||||||||||||||||||||||||||||||||||||
       TTGTTGGGGTTGGGGTTGGTGGTGGTGGTGGTGGTGTGGTGTTGTTGGTG

12700      .    :    .    :    .    :    .    :    .    :
       TTAGTGTTGGTGTTCTGATGTGGTGTTGGTGGTGTTGGTGTTGGTGGTGT
       ||||||||||||||||||||||||||||||||||||||||||||||||||
       TTAGTGTTGGTGTTCTGATGTGGTGTTGGTGGTGTTGGTGTTGGTGGTGT

12750      .    :    .    :    .    :    .    :    .    :
       TGTTGGTGTGGTGGTGTTGGTGTGGTGTTGTTGGTGTTGGTGTTGGTGGT
       ||||||||||||||||||||||||||||||||||||||||||||||||||
       TGTTGGTGTGGTGGTGTTGGTGTGGTGTTGTTGGTGTTGGTGTTGGTGGT

12800      .    :    .    :    .    :    .    :    .    :
       GTTGGTGTTGGTGTGGTGTTGTTGGTGTTGGTGTGGT-------------
       |||||||||||||||||||||||||||||||||||||
       GTTGGTGTTGGTGTGGTGTTGTTGGTGTTGGTGTGGTGTTGGTGTTGGTG


       ...


16000      .    :    .    :    .    :    .    :    .    :
       -------------------------------------------GTGATGA
                                                  |||||||
       TGATGGTAGGACAGTGGTGGTGGTGATGATGGTGGTGGTGAGGGTGATGA


16050      .    :    .    :    .    :    .    :    .    :
       TGGTGGTGGTATTGTGGTAATAGGATGGTGGTGGTAGTATGGTGGTGGTG
       ||||||||||||||||||||||||||||||||||||||||||||||||||
       TGGTGGTGGTATTGTGGTAATAGGATGGTGGTGGTAGTATGGTGGTGGTG

16100      .    :    .    :    .    :    .    :    .    :
       GTGGTAACAGTAGGCATGGTGGTGATTACTATATAACGATGATAACGTTC
       ||||||||||||||||||||||||||||||||||||||||||||||||||
       GTGGTAACAGTAGGCATGGTGGTGATTACTATATAACGATGATAACGTTC

16150      .    :    .    :    .    :    .    :    .    :
       TTGTGGAGGTGACTGTGTCGATGACACACTTGTGTGTCTGATGCCAAGTG
       ||||||||||||||||||||||||||||||||||||||||||||||||||
       TTGTGGAGGTGACTGTGTCGATGACACACTTGTGTGTCTGATGCCAAGTG

16200      .    :    .    :    .    :    .    :    .    :
       CTCCCTGGACTTCTGTTTGCTCCTGGGCTCTTTTCCATCCCAGCTCTTCA
       ||||||||||||||||||||||||||||||||||||||||||||||||||
       CTCCCTGGACTTCTGTTTGCTCCTGGGCTCTTTTCCATCCCAGCTCTTCA
```

Figure S7: Closer inspection of the alignment shown in **Figure S6** reveals the presence of a large insertion that extends up to position 16,104. Realignment of this region[1] resulted in an alignment (shown here in excerpts), in which the gappy region starting at position 12,839 of the original alignment is placed as a single matched block on the 3' end of the deletion. As in **Figure S6**, `REGION155` is shown on the top and `chr8_KI270820v1_alt` on the bottom of the alignment.

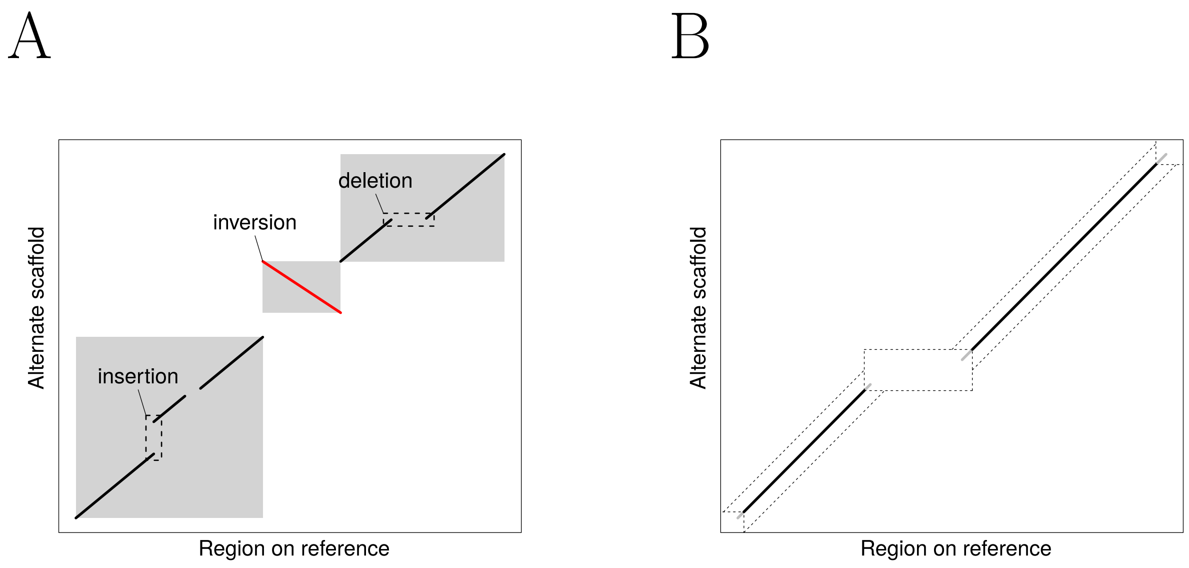

Figure S8: (A) Banded Alignment. M (matching) blocks derived from the NCBI alignment are shown as black and red lines. There is a large inversion in the middle of the alignment (red), and thus the whole alignment is divided into three blocks (in gray) that were considered for the banded chain alignment. In the representation of the alignment in the GFF file, the inversion is encoded as a deletion of the original sequence together with an insertion of the inverted sequence. **(B) Determination of seeds for banded-chain alignment**. The figure shows an excerpt of the alignment in Panel A showing the third matching block/band. The matching (M) blocks of the NCBI alignments are shown as black bars with gray ends. The gray ends are clipped off according to Algorithm **S1**, and the remaining black bars represent the seeds used for the banded-chain[2] alignment. The regions analyzed with the banded alignment are shown as dotted lines.

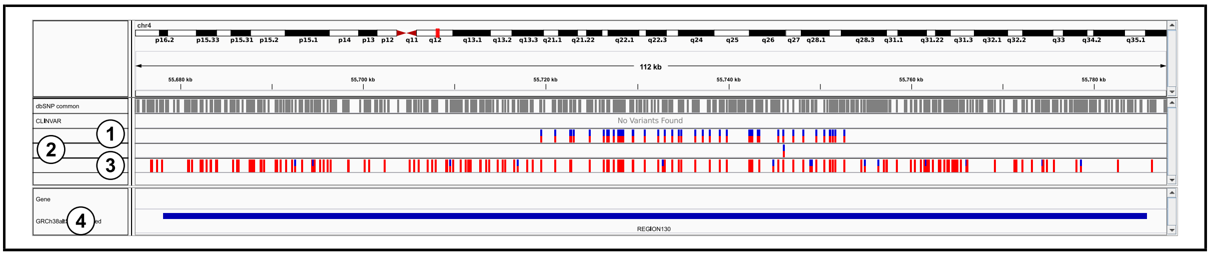

Figure S9: Anchors for alignments The alignments between regions and alternate loci begin with identical anchors, such that the beginning and end portions of the alignments are identical. No ASDPs occur in these regions. Therefore, the analysis described in Algorithm **S2** is limited to the alignment region and called variants ③ located from the first to the last ASDP in any given region. The Figure shows an IGV screenshot of ④ REGION130 (chr4:55,678,095-55,785,754). The ASDP-containing portion ① & ② of the region comprises chr4:55,719,527-55,752,683.

A

```
69100      .    :    .    :    .    :    .    :    .    :
       TTTTGGTAATAGTGTAGGGACCAGATTGCTGGTGGGAAAATTGGGGAAGG
       ||||||||||||||||||||| ||||||||||||||||||||||||||||
       TTTTGGTAATAGTGTAGGGACGAGATTGCTGGTGGGAAAATTGGGGAAGG

69150      .    :    .    :    .    :    .    :    .    :
       AGGAATCAAATTTTAAGAGACTGTTCTAGTAATCAGGGTGAAAACTTAGA
       ||||||||   ||||||||||||||||||||||||||||||||||||| |
       AGGAATCA---TTTAAGAGACTGTTCTAGTAATCAGGGTGAAAACTTATA
```

B

```
#CHROM  POS       ID     REF    ALT    QUAL    FILTER  INFO  FORMAT  SAMPLE
chr8    39163439  .      C      G      40      PASS  AL=chr8_KI270822v1_alt;RE=ADAM5;RP=69121  GT 0|1
chr8    39163475  .      AAAT   A      40      PASS  AL=chr8_KI270822v1_alt;RE=ADAM5;RP=69158  GT 0|1
chr8    39163516  .      G      T      40      PASS  AL=chr8_KI270822v1_alt;RE=ADAM5;RP=69198  GT 0|1
```

Figure S10: Representation of ASDP-associated variants in the VCF file used by ASDPex to record ASDPs. **(A)** The alignment displays single-nucleotide mismatches at position `69,122` and `69,199` and a three-nucleotide deletion in the scaffold from `69,159-69,161`. **(B)** An excerpt of the VCF file representing the variants in Panel A. ASDPex stores the fields AL (alternate locus), here `chr8_KI270822v1_alt`, RE (region), here `ADAM5`, and RP (region position) in the `INFO` column.

```
...
##FILTER=<ID=ASDP,Description="Filtered due to a more likely alternative scaffold">
##INFO=<ID=ALTGENOTYPE,Number=A,Type=String,Description="most likely alternate scaffold replacement
genotype">
##INFO=<ID=ALTLOCUS,Number=A,Type=String,Description="most likely alternate scaffold id replacement">
...
#CHROM  POS     ID    REF   ALT   QUAL     FILTER  INFO          FORMAT  SAMPLE
chr1    72126   .     G     C     1539.99  ASDP    ALTGENOTYPE=HOM_VAR;ALTLOCUS=chr1_KI270760v1_alt;...  GT      1/1
...
chr2    861114  .     C     T     620.70   ASDP    ALTGENOTYPE=HET;ALTLOCUS=chr2_GL383522v1_alt;...  GT      0/1
```

Figure S11: Modifications to the VCF files after annotation with ASDPex. At the top the new descriptive header lines are shown, defining the new additional flags for FILTER and INFO in the VCF entries. In addition two VCF entries annotated as ASDP-associated-variants are shown. The first is a homozygous variant of a region also called as probable homozygous alternate locus. The second a heterozygous ASDP-associated-variant in a heterozygous annotated region.

A

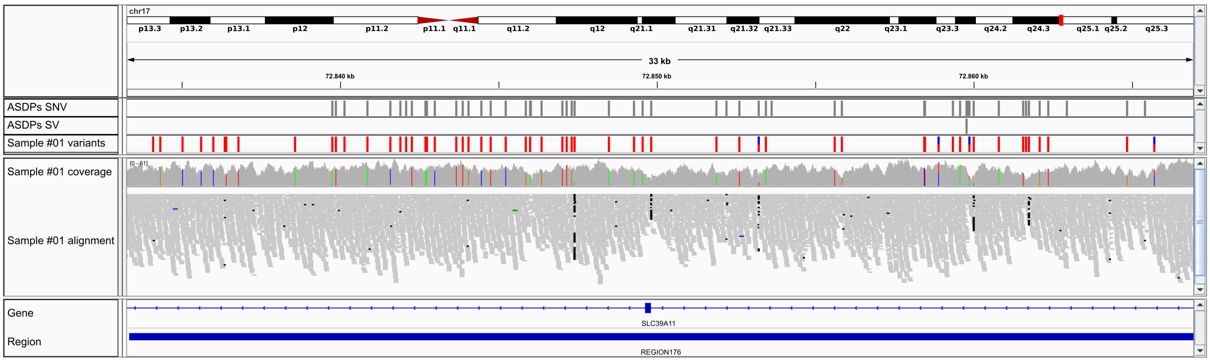

B

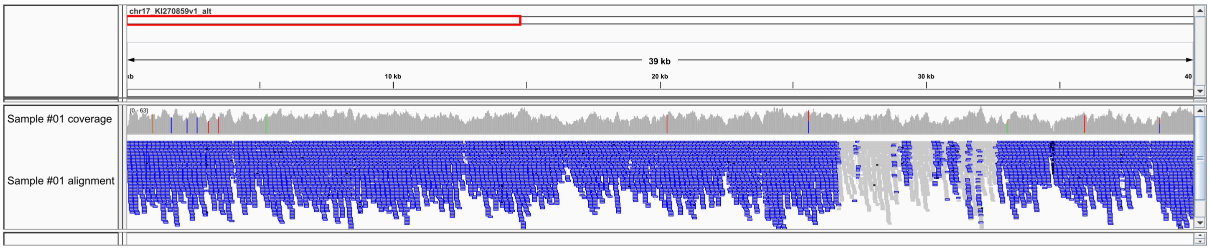

Figure S12: REGION176 vs. KI270859.1. **(A)** Sample #01 shows numerous homozygous ASDP-associated variants in region REGION176 (72,833,239-72,866,965 on chromosome 17). **(B)** The corresponding region on the alternate locus KI270859.1 displays many fewer discrepancies between aligned reads and ALT-HAP. One can infer that sample #01 is homozygous for KI270859.1.

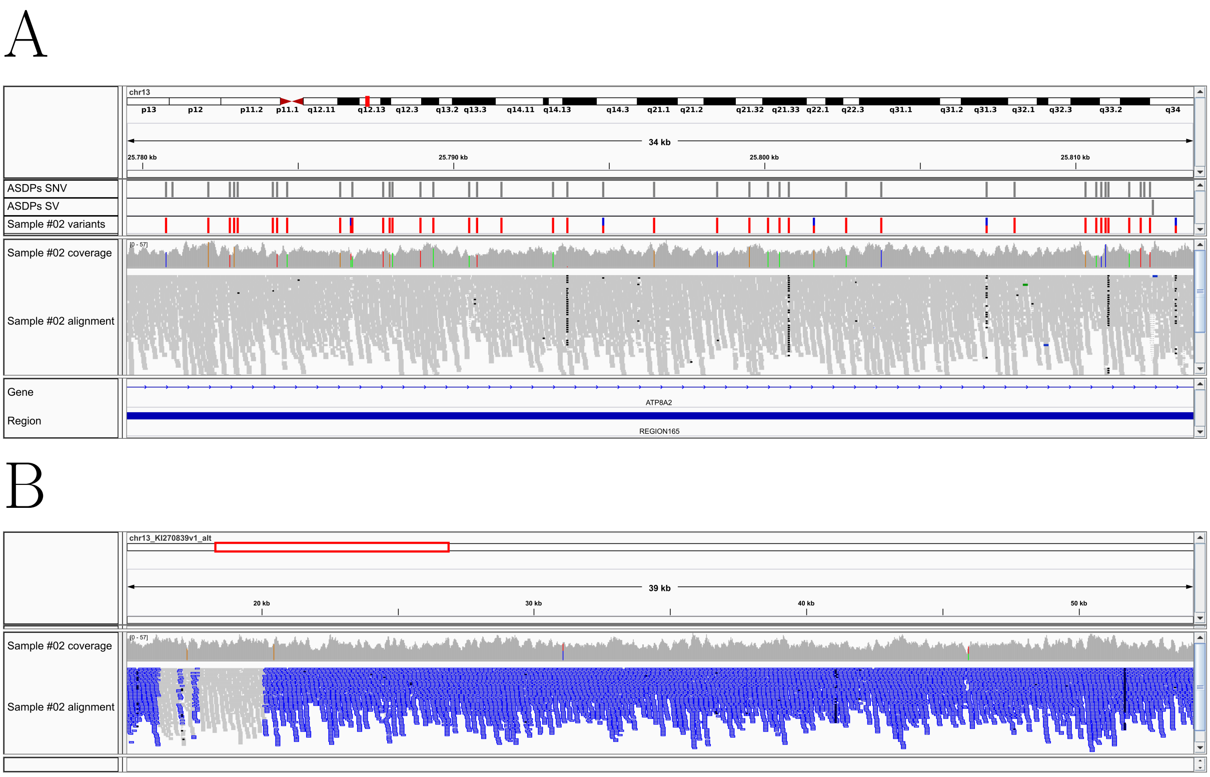

Figure S13: REGION165 vs. KI270839.1. **(A)** Sample #02 shows numerous homozygous ASDP-associated variants in REGION165 (25,779,488-25,813,821 on chromosome 13) **(B)** The corresponding region on the alternate locus KI270839.1 displays many fewer discrepancies between aligned reads and ALT-HAP. One can infer that sample #02 is homozygous for KI270839.1.

A

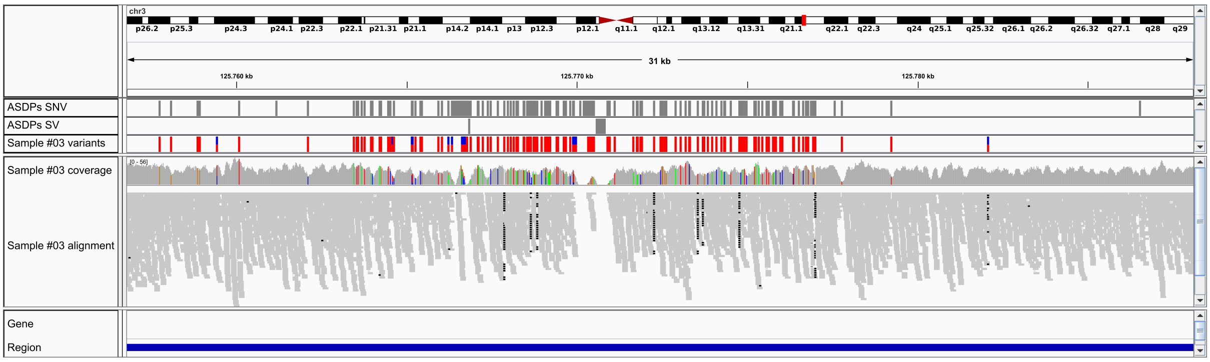

B

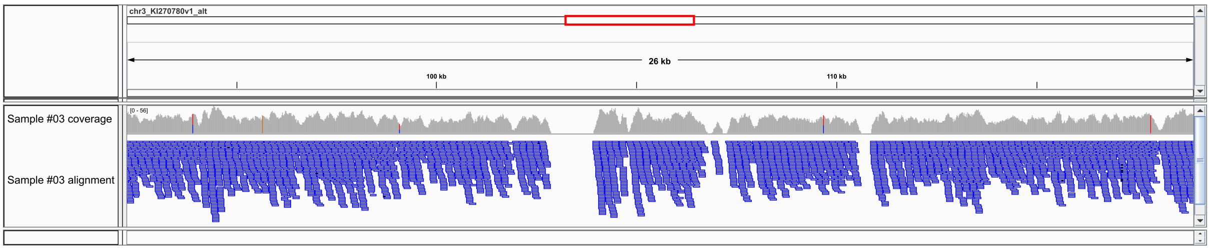

Figure S14: REGION123 vs. KI270780.1. **(A)** Sample #03 shows numerous homozygous ASDP-associated variants in REGION123 (125,756,772-125,788,115 on chromosome 3) **(B)** The corresponding region on the alternate locus KI270780.1 displays many fewer discrepancies between aligned reads and ALT-HAP. One can infer that sample #03 is homozygous for KI270780.1.

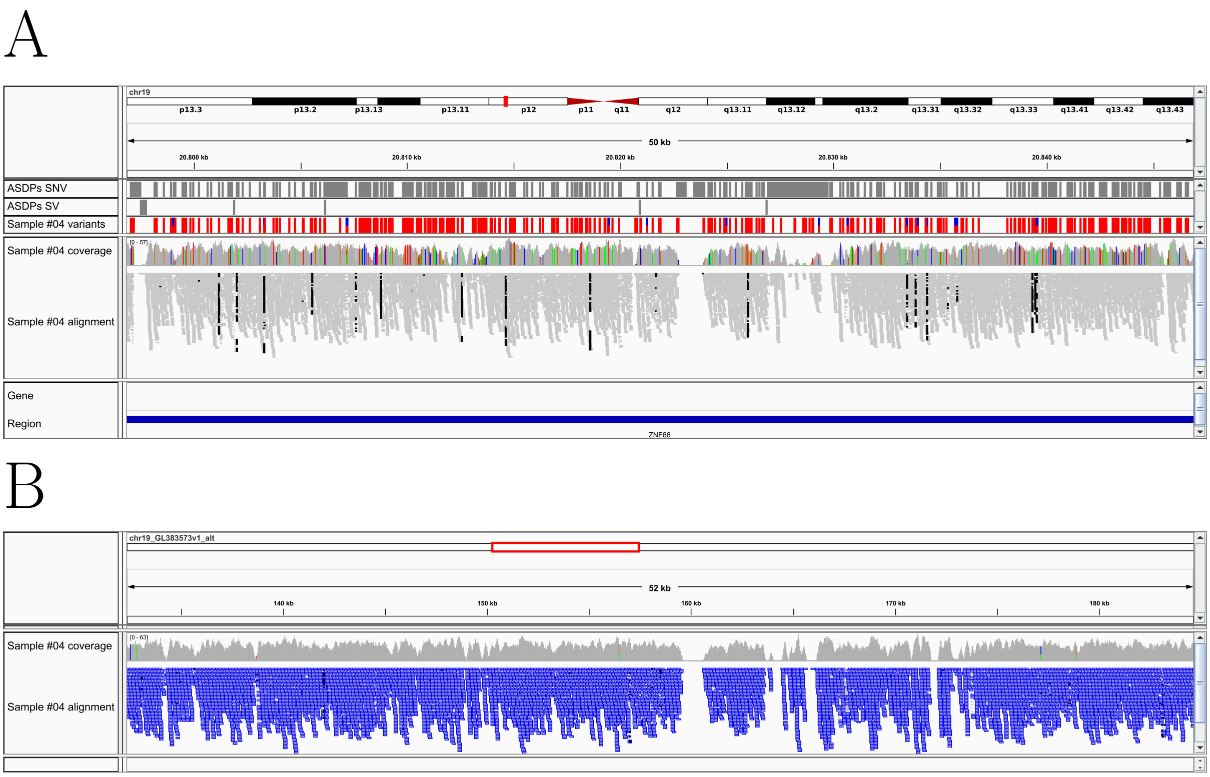

Figure S15: ZNF66 vs. GL383573.1. **(A)** Sample #04 shows numerous homozygous ASDP-associated variants in ZNF66 (20,796,825-20,846,912 on chromosome 19) **(B)** The corresponding region on the alternate locus GL383573.1 displays many fewer discrepancies between aligned reads and ALT-HAP. One can infer that sample #04 is homozygous for GL383573.1.

A

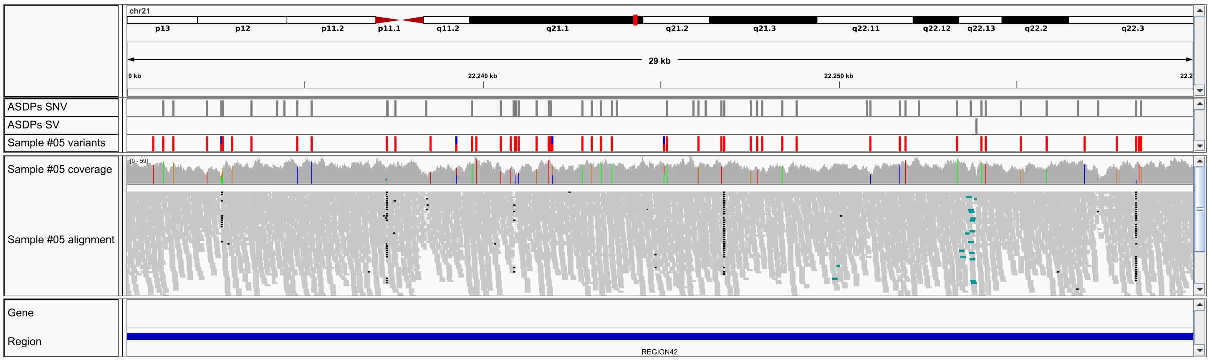

B

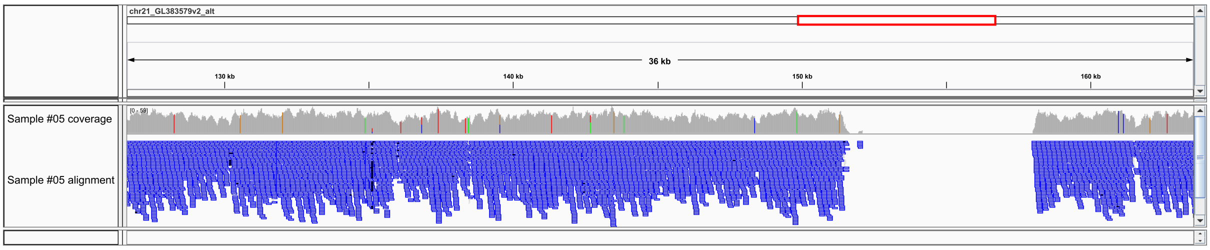

Figure S16: REGION42 vs. GL383579.2. **(A)** Sample #05 shows numerous homozygous ASDP-associated variants in REGION42 (22,229,996-22,259,971 on chromosome 21) **(B)** The corresponding region on the alternate locus GL383579.2 displays many fewer discrepancies between aligned reads and ALT-HAP. One can infer that sample #05 is homozygous for GL383579.2.

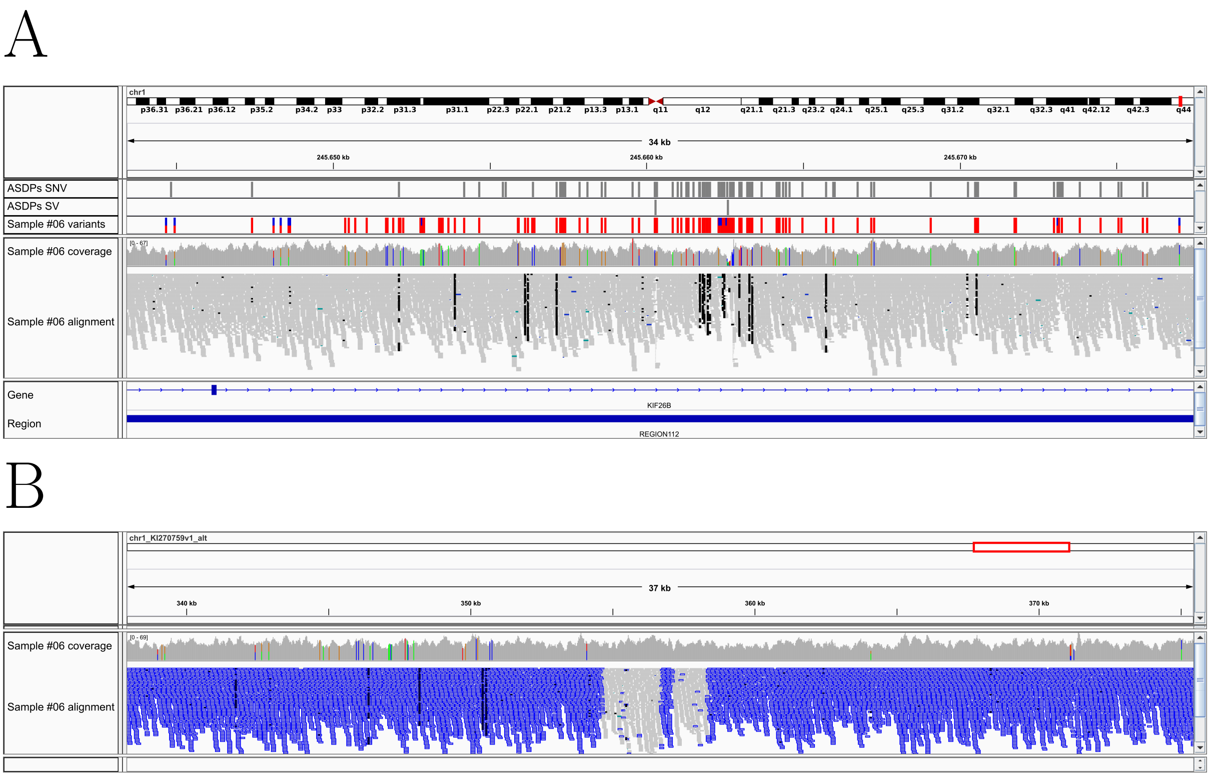

Figure S17: REGION112 vs. KI270759.1. **(A)** Sample #06 shows numerous homozygous ASDP-associated variants in REGION112 (245,643,406-245,677,474 on chromosome 1) **(B)** The corresponding region on the alternate locus KI270759.1 displays many fewer discrepancies between aligned reads and ALT-HAP. One can infer that sample #06 is homozygous for KI270759.1.

A

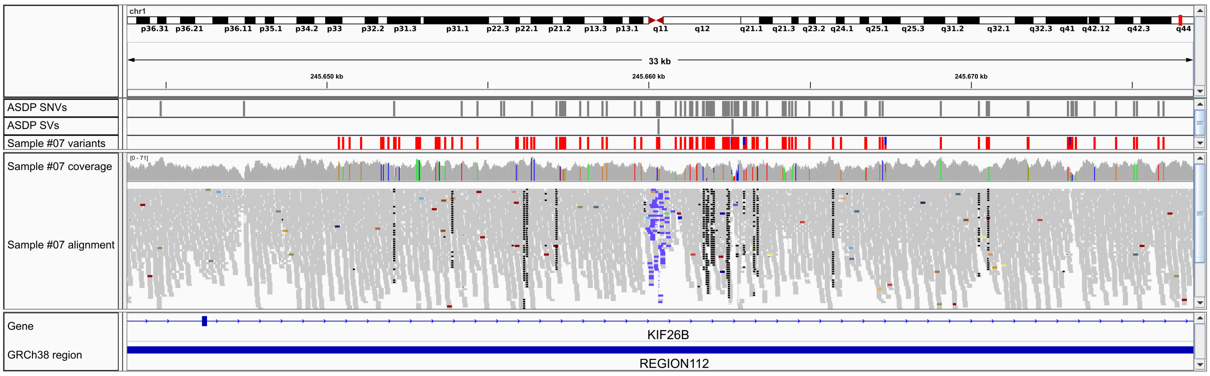

B

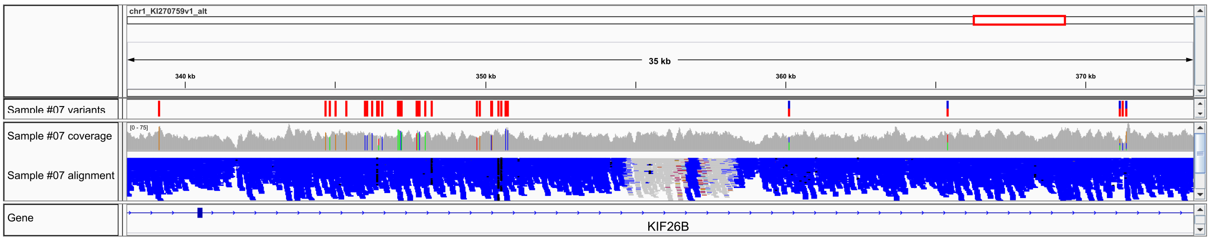

Figure S18: REGION112 vs. KI270759.1. **(A)** Sample #07 shows numerous homozygous variants in REGION112 (245,643,787-245,676,929 on chromosome 1). Starting from 245, 680, 000 most are ASDP-associated variants indicating a recombination between the KI270759.1 ALT-HAP and another (undefined) sequence. **(B)** The corresponding region on the alternate locus KI270759.1 displays many fewer discrepancies between aligned reads and ALT-HAP in the second half. One can infer that sample #07 is homozygous for this part for KI270759.1.

A

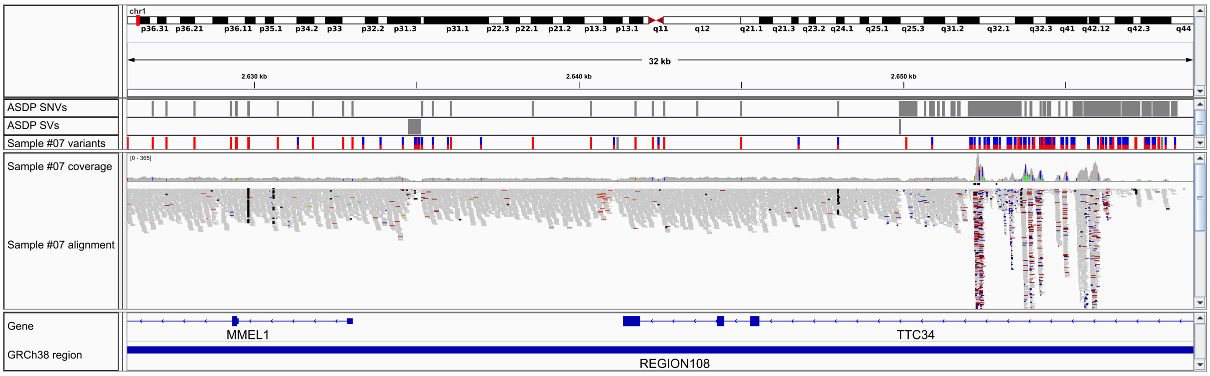

B

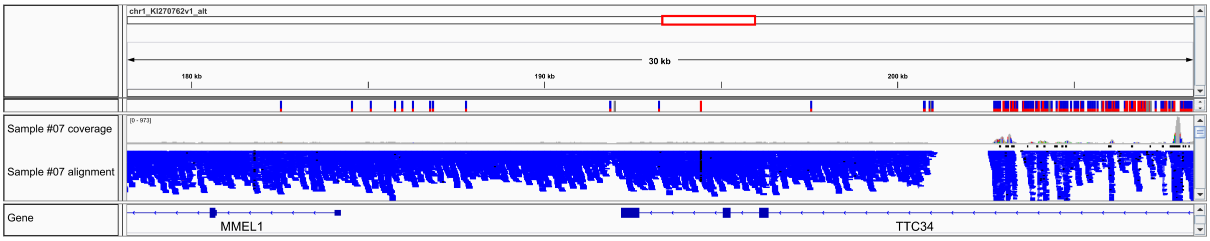

Figure S19: REGION108 vs. KI270759.1. **(A)** Sample #07 shows numerous homozygous ASDP-associated variants in REGION108 (2,626,066-2,658,966 on chromosome 1) up to $2,645,000$, which suggests a recombination between the KI270762.1 ALT-HAP and another (undefined) sequence. **(B)** The corresponding region on the alternate locus KI270762.1 displays many fewer discrepancies between aligned reads and ALT-HAP in the part with the ASDP-associated variants and One can infer that sample #07 is homozygous for KI270762.1 for part of the region.

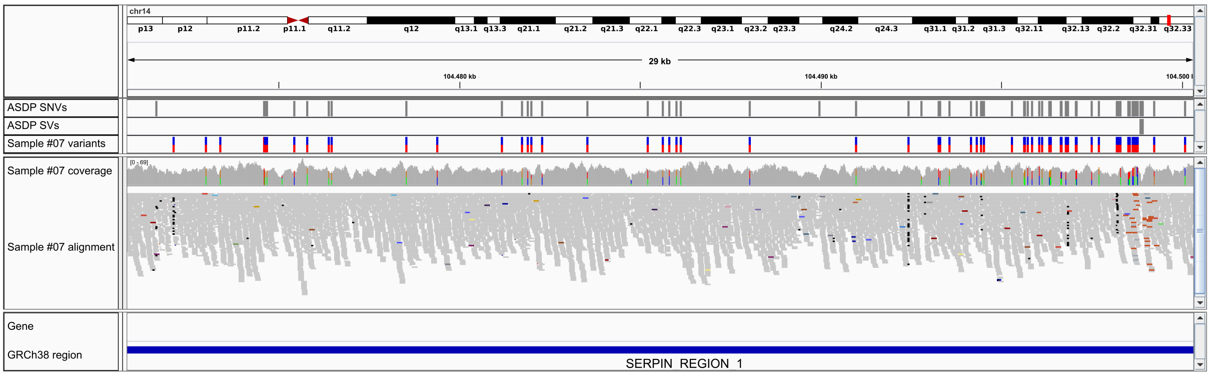

Figure S20: SERPIN_REGION_1 vs. KI270845.1. Sample #07 shows numerous heterozygous ASDP-associated variants origin from the alignment against KI270845.1 in SERPIN_REGION_1 (104,470,796-104,500,326 on chromosome 14). One can infer that sample #07 is heterozygous for KI270845.1.

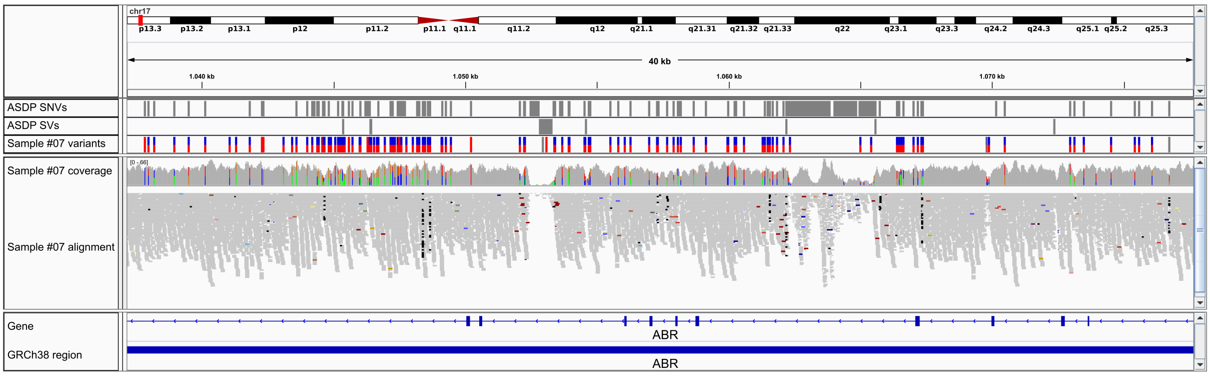

Figure S21: ABR vs. KI270910.1. Sample #07 shows numerous heterozygous ASDP-associated variants origin from the alignment against KI270910.1 in ABR (1,037,273-1,077,824 on chromosome 17). One can infer that sample #07 is heterozygous for KI270910.1.

A

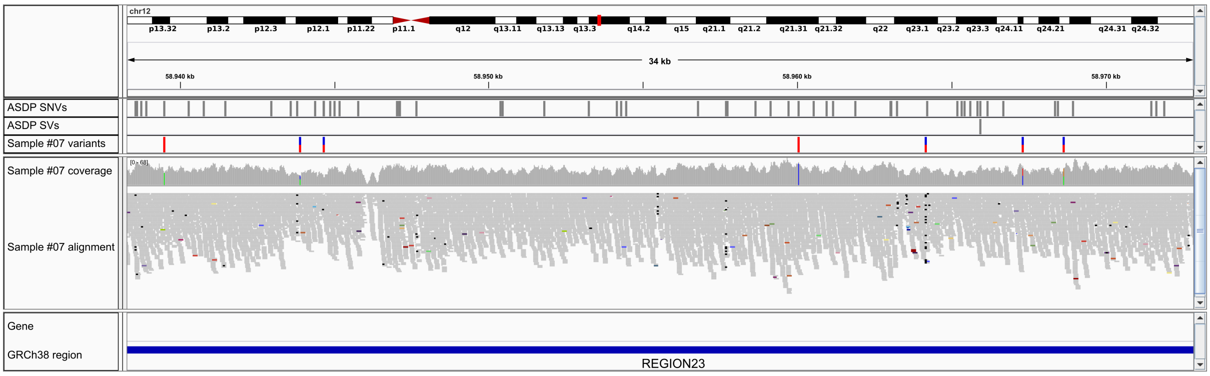

B

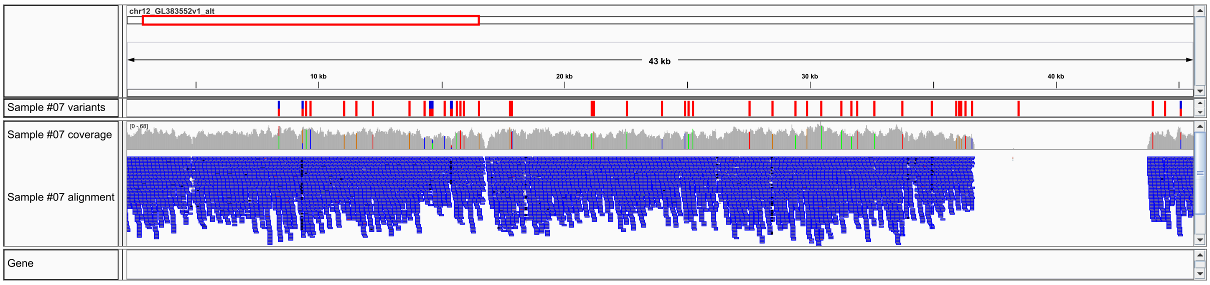

Figure S22: REGION23 vs. GL383552.1. These two figures demonstrate the overlap between known ASDPs and called variants for a randomly chosen region which was not called as homozygous or heterozygous ALT-HAP. **A** Sample #07 shows nearly no ASDP-associated variants in REGION23 (58,938,274-58,972,856 on chromosome 12). **B** The corresponding region on the alternate locus GL383552.1 shows several homozygous ASDP-associated variantsȮne can infer that sample #07 is homozygous for REF-HAP.

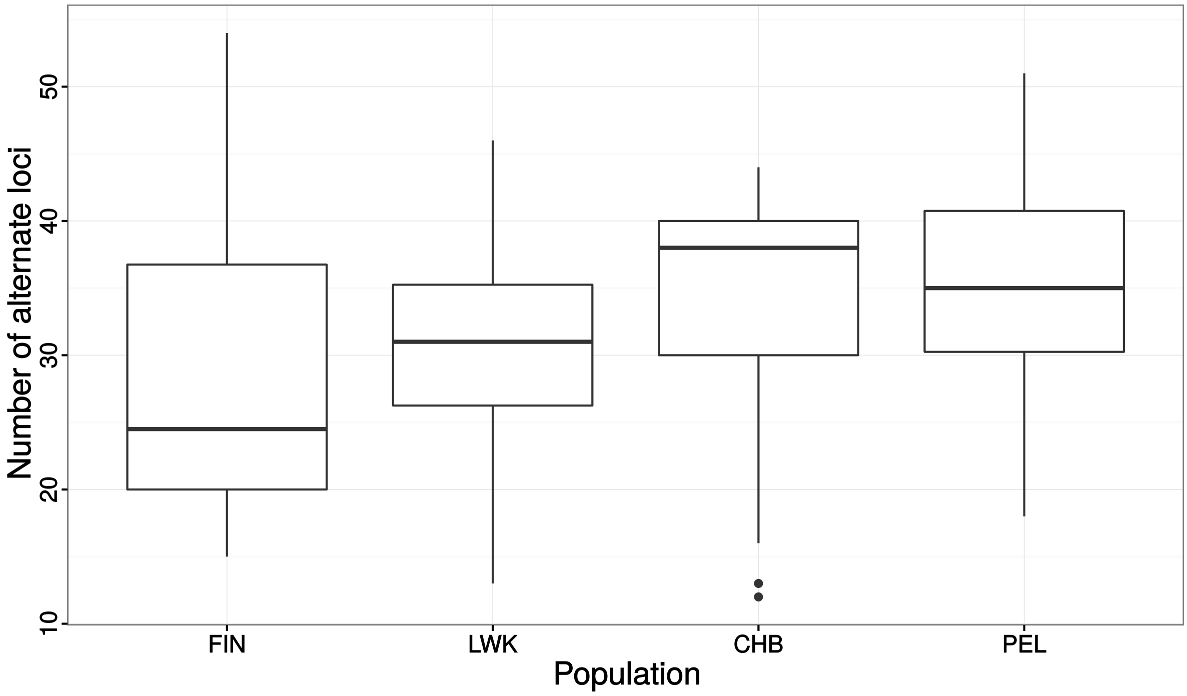

Figure S23: Number of annotated alternate loci per population. For four populations each 30 individuals the number of annotated alternate loci were counted. **FIN**: Finnish in Finland; **LWK**: Luhya in Webuye, Kenya; **CHB**: Han Chinese in Beijing, China; **PEL**: Peruvians from Lima, Peru.

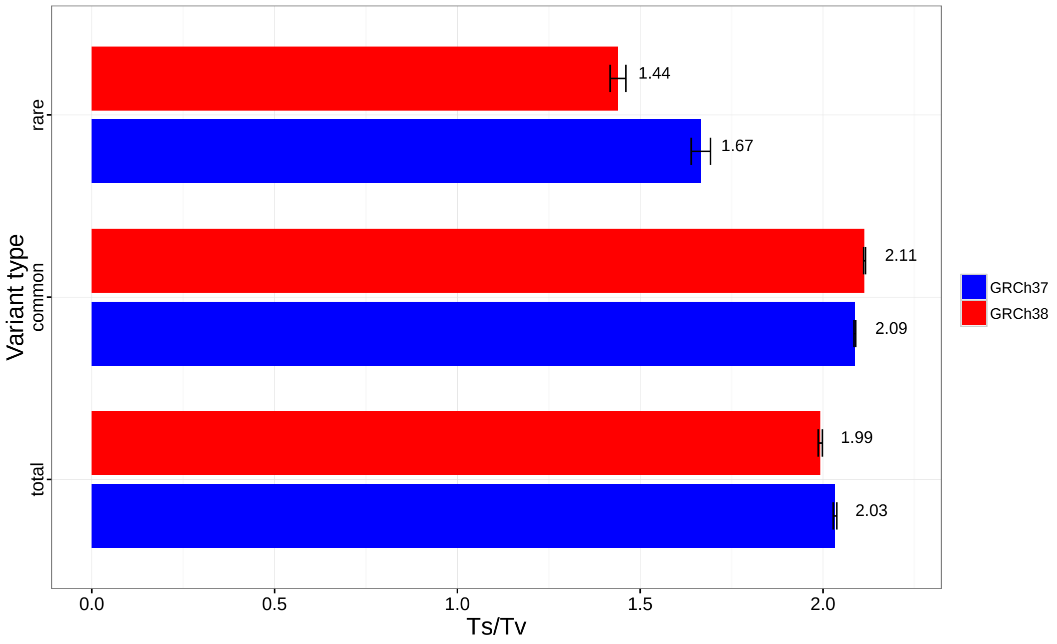

Figure S24: Ts/Tv ratio for both genome builds. The Ts/Tv ratio for GRCh37 (blue, bottom) and GRCh38 (red, top) for high quality variants (Phred-score $\geqq$ 30) is shown for single nucleotide substitutions called as *rare*, for *common* SNPs (dbSNP 146), and for all SNPs (*total*).

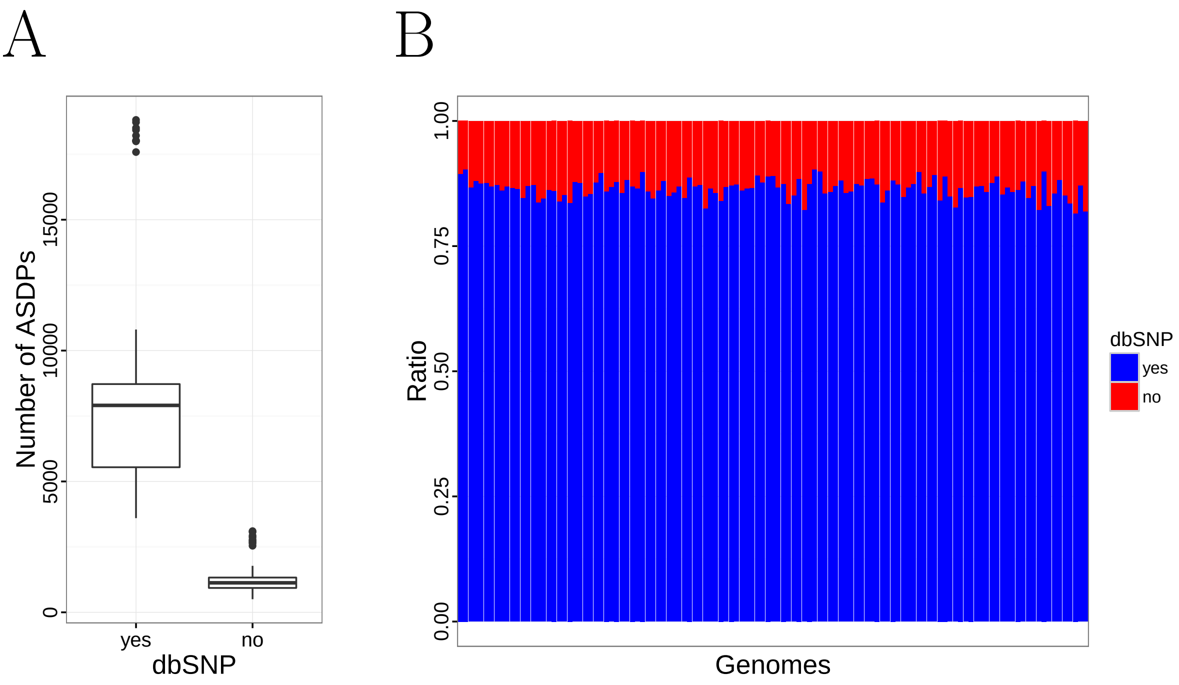

Figure S25: In-house ASDPs with dbSNP annotation. (**A**) The plot shows the distribution of the ASDP variants in our in-house genomes that are listed in dbSNP. In (**B**) the ratio of ASDP variants with dbSNP entries within genomes is plotted against those without dbSNP entries. On average 13.48% of the ASDP-associated variants are not listed in dbSNP.

A

```
2:1110:32373:30844    99   chr7   148380548   60   87M1I63M   =   148380991    594   ...
SA:Z:chr7_KI270808v1_alt,162211,+,151M,17,1;  ...
2:1110:32373:30844   147   chr7   148380991   60       151M   =   148380548   -594   ...
SA:Z:chr7_KI270808v1_alt,162655,-,151M,4,17;   ...
```

B

```
2:1110:32373:30844      2147    chr7_KI270808v1_alt     162211  60      151M  ...
2:1110:32373:30844      2195    chr7_KI270808v1_alt     162655  60      151M   ...
```

C

	**Left alignment**	**Right alignment**
	Primary alignment	
Sample	sampleP	sampleP
Read length	151bp	151bp
Mapping Quality	MAPQ 60	MAPQ 60
Reference span	chr7:148.380.548-148.380.697 (+)	chr7:148.380.991-148.381.141 (-)
Cigar	87M1I63M	151M
Location	chr7:148.380.640	
Base	T @ QV 42	
Mate is mapped	yes	yes
Mate start	chr7:148.380.991 (-)	chr7:148.380.548 (+)
Insert size	594	-594
	First in pair	Second in pair
Pair orientation	F1R2	F1R2
	Supplementary alignment	
Mapping Quality	MAPQ 60	MAPQ 60
Reference span	chr7_KI270808v1_alt:162.211-162.360 (+)	chr7_KI270808v1_alt:162.655-162.804 (-)
Cigar	151M	151M
Mate is mapped	yes	yes
Mate start	chr7_KI270808v1_alt:162.655 (-)	chr7_KI270808v1_alt:162.211 (+)
Insert size	595	-595
	First in pair	Second in pair
Pair orientation	F1R2	F1R2

Figure S26: SAM format representation of the supplementary reads. The read alignment representation is shown for one read pair spanning a homozygous ASDP-associated insertion from **Figure 3**. (**A**) Shortened representation of the read alignment in SAM format. Notice the sixth column with the CIGAR string for the alignment and the column with the supplementary alignment information (SA:Z). The first mate pair read indicates an insertion of a *T* which is not present in the supplementary alignment. (**B**) The information from the SAM formated lines for the mate pair and (**C**) the primary and supplementary alignment.

Field	Example	Explanation
alt_asm_name	ALT_REF_LOCI_1	Alternate assembly name
prim_asm_name	Primary Assembly	Primary assembly name
alt_scaf_name	HSCHR1_1_CTG3	Name of alternate scaffold in GenBank file referred to by NT_187515.1
alt_scaf_acc	NT_187515.1	Accession number of the alternate scaffold
parent_type	CHROMOSOME	Type of sequence to which this alt scaffold is assigned
parent_name	1	The name (i.e., chromosome 1)
parent_acc	NC_000001.11	Accession number of the parent sequence (corresponds to the information in the file chr_accessions_GRCh38.p2)
region_name	REGION108	Name of the genomic region to which this alternate scaffold is assigned (corresponds to the information in the file genomic_regions_definitions.txt; in this example, the latter file specifies that REGION108 is located on NC_000001.11 with start position 2448811 and stop position 2791270).
ori	+	One of "+", "-", or "b"
alt_scaf_start	1	Start position of alignment with respect to the sequence of the alt locus.
alt_scaf_stop	354444	Stop position of alignment with respect to the sequence of the alt locus. In this example, the alignment includes the entire sequence of NT_187515.1, which is 354444 bp in length
parent_start	2448811	Start position of alignment on parent
parent_stop	2791270	Stop position of alignment on parent. Here, the positions correspond exactly with the positions for REGION108 in the genomic_regions_definitions.txt file.
alt_start_tail	0	This is the length of an insertion as compared to the reference sequence. For instance, NT_187517.1 has an alt_start_tail of 20,632 nucleotides and the alignment with the corresponding reference begins at position 20,633 of the sequence in NT_187517.1
alt_stop_tail	0	Analogous to alt_start_tail but the insertion is at the end of the scaffold sequence; see for instance NT_187557.1.

Table S1: The fields of the "alt_scaffold_placement.txt" files.

Range	Number	Percentage
<100 kb	27	15.2%
100-200 kb	89	50.0%
200-500 kb	43	24.1%
500 kb-2 Mb	13	7.3%
>2 Mb	6	3.4%

Table S2: Size distribution of the 178 REF-HAP regions of the GRCh38 genome build.

<table>
<tr><th>Impact</th><th>Counts</th><th>SO-term</th></tr>
<tr><td>HIGH</td><td>1136</td><td>mnv</td></tr>
<tr><td></td><td>168</td><td>stop_gained</td></tr>
<tr><td></td><td>126</td><td>stop_lost</td></tr>
<tr><td></td><td>81</td><td>internal_feature_elongation|complex_substitution</td></tr>
<tr><td></td><td>79</td><td>frameshift_elongation</td></tr>
<tr><td></td><td>75</td><td>frameshift_variant</td></tr>
<tr><td></td><td>68</td><td>feature_truncation|complex_substitution</td></tr>
<tr><td></td><td>47</td><td>frameshift_truncation</td></tr>
<tr><td></td><td>22</td><td>mnv|stop_gained</td></tr>
<tr><td></td><td>14</td><td>splice_donor_variant|non_coding_transcript_intron_variant</td></tr>
<tr><td></td><td>9</td><td>frameshift_truncation|complex_substitution|splice_donor_variant</td></tr>
<tr><td></td><td>9</td><td>mnv|splice_region_variant</td></tr>
<tr><td></td><td>8</td><td>frameshift_truncation|complex_substitution|splice_acceptor_variant</td></tr>
<tr><td></td><td>8</td><td>splice_donor_variant|coding_transcript_intron_variant</td></tr>
<tr><td></td><td>7</td><td>start_lost|missense_variant</td></tr>
<tr><td></td><td>6</td><td>splice_acceptor_variant|coding_transcript_intron_variant</td></tr>
<tr><td></td><td>3</td><td>feature_truncation|complex_substitution|splice_acceptor_variant</td></tr>
<tr><td></td><td>3</td><td>splice_acceptor_variant|non_coding_transcript_intron_variant</td></tr>
<tr><td></td><td>3</td><td>stop_lost|missense_variant</td></tr>
<tr><td></td><td>2</td><td>start_lost</td></tr>
<tr><td></td><td>1</td><td>complex_substitution|stop_lost</td></tr>
<tr><td></td><td>1</td><td>feature_truncation|complex_substitution|splice_donor_variant</td></tr>
<tr><td></td><td>1</td><td>feature_truncation|complex_substitution|splice_region_variant</td></tr>
<tr><td></td><td>1</td><td>frameshift_elongation|complex_substitution|splice_acceptor_variant</td></tr>
<tr><td></td><td>1</td><td>frameshift_truncation|splice_acceptor_variant</td></tr>
<tr><td></td><td>1</td><td>frameshift_truncation|stop_lost</td></tr>
<tr><td></td><td>1</td><td>frameshift_variant|complex_substitution|splice_donor_variant</td></tr>
<tr><td></td><td>1</td><td>frameshift_variant|complex_substitution|stop_lost</td></tr>
<tr><td></td><td>1</td><td>frameshift_variant|splice_region_variant</td></tr>
<tr><td></td><td>1</td><td>internal_feature_elongation|complex_substitution|splice_region_variant</td></tr>
<tr><td></td><td>1</td><td>splice_acceptor_variant|5_prime_utr_variant</td></tr>
<tr><td></td><td>1</td><td>splice_acceptor_variant|non_coding_transcript_exon_variant</td></tr>
<tr><td></td><td>1</td><td>stop_gained|splice_region_variant</td></tr>
<tr><td>MODERATE</td><td>5381</td><td>missense_variant</td></tr>
<tr><td></td><td>71</td><td>missense_variant|splice_region_variant</td></tr>
<tr><td></td><td>321</td><td>splice_region_variant|coding_transcript_intron_variant</td></tr>
<tr><td></td><td>59</td><td>splice_region_variant|non_coding_transcript_intron_variant</td></tr>
<tr><td></td><td>45</td><td>splice_region_variant|synonymous_variant</td></tr>
<tr><td></td><td>32</td><td>splice_region_variant|non_coding_transcript_exon_variant</td></tr>
<tr><td></td><td>31</td><td>disruptive_inframe_deletion</td></tr>
<tr><td></td><td>15</td><td>disruptive_inframe_insertion|direct_tandem_duplication</td></tr>
<tr><td></td><td>14</td><td>disruptive_inframe_insertion</td></tr>
<tr><td></td><td>12</td><td>splice_region_variant|5_prime_utr_variant</td></tr>
<tr><td></td><td>11</td><td>inframe_deletion</td></tr>
<tr><td></td><td>3</td><td>inframe_insertion</td></tr>
<tr><td></td><td>2</td><td>splice_region_variant|3_prime_utr_variant</td></tr>
<tr><td></td><td>1</td><td>inframe_insertion|direct_tandem_duplication</td></tr>
<tr><td>LOW</td><td>65765</td><td>coding_transcript_intron_variant</td></tr>
<tr><td></td><td>14482</td><td>non_coding_transcript_intron_variant</td></tr>
<tr><td></td><td>2251</td><td>synonymous_variant</td></tr>
<tr><td></td><td>1963</td><td>3_prime_utr_variant</td></tr>
<tr><td></td><td>1713</td><td>non_coding_transcript_exon_variant</td></tr>
<tr><td>s</td><td>1276</td><td>5_prime_utr_variant</td></tr>
<tr><td></td><td>7</td><td>stop_retained_variant|synonymous_variant</td></tr>
<tr><td>MODIFIER</td><td>125978</td><td>intergenic_variant</td></tr>
<tr><td></td><td>5762</td><td>downstream_gene_variant</td></tr>
<tr><td></td><td>5251</td><td>upstream_gene_variant</td></tr>
</table>

Table S3: Sequence ontology categories of the ASDPs identified in the GRCh38.p2 genome release. The curated dataset of valid ASDPs (collapsed, window 50b mismatches 10) was annotated using Jannovar[3] (v. 0.16) and the RefSeq GRCh38.p2 annotations. Variant categories (SO-term(s)[4] shown in the third column) are sorted according to their putative impact (first column) then total counts (second column) of annotated ASDPs.

Table S4: GWAS Hits that Overlap ASDPs The GWAS catalog was downloaded (2016-02-01). Each GWAS hit was analyzed for overlap with the curated dataset of valid ASDPs.

Chromosome	Position	dbSNP ID	Reference	Alt allele
1	155225189	rs2049805	T	C
1	243852691	rs4430311	C	T
11	589564	rs4963128	T	C
11	1100037	rs7934606	C	T
11	3007859	rs7481584	G	A
11	3015094	rs739401	C	T
11	56698623	rs1397048	C	T
12	11063716	rs2708377	C	T
12	11126493	rs1031391	C	G
12	28040056	rs7964407	G	A
15	28111713	rs1129038	C	T
15	28120472	rs12913832	A	G
15	28268218	rs916977	T	C
15	28285036	rs1667394	C	T
15	28760947	rs8033165	C	T
15	29132552	rs2636061	A	G
15	29596192	rs1471225	T	C
15	29901265	rs711355	T	C
15	30606129	rs143536437	C	T
15	31171175	rs7169523	G	A
15	31501727	rs7164569	A	G
15	32702555	rs4779584	T	C
16	489140	rs2038227	C	A
16	15493640	rs34792	C	G
16	16029602	rs924135	A	T
16	16033378	rs2062541	G	A
16	16034885	rs246234	C	G
16	16162910	rs3213473	T	G
16	16636869	rs9937036	GATAT	G
16	16864058	rs7186128	G	A
17	189133	rs7217319	T	C
17	36031260	rs712046	C	T
17	37666305	rs6607284	T	C
17	37736310	rs2005705	G	A
17	37736525	rs757210	C	T
17	37738049	rs4430796	A	G
17	37741165	rs7501939	C	T
17	41094387	rs4006360	C	T
17	45436075	rs11012	C	T
17	45439036	rs17631303	A	G
17	46710944	rs183211	G	A
17	46751565	rs199533	G	A
17	46779275	rs199515	G	C
17	46781778	rs415430	C	T
17	46788073	rs2074404	T	G
17	46788237	rs199498	T	C
18	78655450	rs66591657	A	G
19	54109321	rs254262	G	A
19	54172738	rs2576452	T	C
19	54173495	rs8736	T	C
19	54193224	rs7595	T	C
19	54288907	rs386000	C	G

continued on the next page

Chromosome	Position	dbSNP ID	Reference	Alt allele
19	54293995	rs103294	T	C
19	54404500	rs11084337	C	T
19	54871595	rs11672983	G	A
2	1404043	rs11675434	C	T
2	1413472	rs2071403	A	G
2	1434216	rs1514687	T	G
22	35729217	rs2016586	T	G
22	42130692	rs1065852	G	A
22	42207808	rs6002655	C	T
22	42225997	rs5758659	C	T
4	68726064	rs293428	A	G
4	189616909	rs13145041	A	G
5	29040014	rs488884	T	G
5	33879687	rs1364044	C	T
6	28744470	rs115329265	A	G
6	28808340	rs4324798	G	A
6	28948475	rs4947339	C	T
6	29116455	rs3129109	T	C
6	29212944	rs9257616	G	A
6	29387425	rs3094548	G	C
6	29388554	rs9257809	A	G
6	29466637	rs4713226	G	A
6	29639269	rs3095267	GG	CA
6	29643654	rs29232	C	T
6	29658544	rs2252711	T	C
6	29702484	rs3129055	A	G
6	29734733	rs2523395	G	A
6	29737882	rs2523393	A	G
6	29755384	rs9258260	C	T
6	29860883	rs2523822	A	G
6	29881842	rs2523809	G	T
6	29931900	rs2524005	G	A
6	29938914	rs2860580	A	G
6	29950322	rs2517713	G	T
6	29952555	rs9260489	T	G
6	29954963	rs16896742	A	G
6	29956061	rs2571391	A	C
6	29966386	rs6935053	A	C
6	29967473	rs3893464	G	A
6	29974166	rs2523946	C	T
6	29974306	rs3823355	C	T
6	29975290	rs6904029	G	A
6	30002812	rs7758512	T	G
6	30006148	rs4313034	C	T
6	30049294	rs6917603	T	C
6	30057726	rs259919	G	A
6	30064745	rs8321	A	C
6	30105999	rs115457135	G	A
6	30108087	rs2023472	A	G
6	30197496	rs2523722	C	T
6	30206354	rs2021722	C	T
6	30211645	rs2844775	G	A
6	30256461	rs116624347	G	T
6	30345563	rs6986	G	C
6	30569829	rs3132613	C	G
6	30711851	rs3094093	T	A

continued on the next page

Chromosome	Position	dbSNP ID	Reference	Alt allele
6	30768374	rs12526186	C	T
6	30769709	rs3094117	A	C
6	30799168	rs4587207	A	G
6	30806580	rs3130783	G	A
6	30814225	rs886424	C	T
6	30830214	rs7749924	C	T
6	30831622	rs9501030	T	A
6	30880476	rs7756521	T	C
6	30908375	rs1052693	C	T
6	30945681	rs3132581	G	A
6	30952347	rs3132580	G	A
6	31008442	rs28360974	G	A
6	31034839	rs4248154	C	T
6	31039078	rs2844665	T	C
6	31049201	rs2251830	C	A
6	31050630	rs2517532	A	G
6	31050769	rs2523864	C	T
6	31057031	rs9262632	A	G
6	31062345	rs2517510	T	G
6	31090563	rs3130544	C	A
6	31101750	rs9263567	T	A
6	31106253	rs6457327	A	C
6	31113428	rs2233956	T	C
6	31125810	rs3815087	G	A
6	31129524	rs3130559	C	T
6	31133897	rs3130564	C	T
6	31138491	rs3130573	A	G
6	31139410	rs1265093	G	A
6	31139481	rs2285803	T	C
6	31143579	rs9263739	C	T
6	31150242	rs1265112	T	C
6	31150734	rs130067	T	G
6	31161533	rs7750641	C	T
6	31162816	rs1419881	G	A
6	31167111	rs3130931	T	C
6	31168676	rs3130501	A	G
6	31168937	rs3132524	T	C
6	31171675	rs879882	T	C
6	31172270	rs1265159	G	A
6	31174468	rs3094188	C	A
6	31175805	rs3131018	A	C
6	31188008	rs1265181	G	C
6	31202751	rs9263871	A	G
6	31216419	rs3869109	A	G
6	31218249	rs9263963	A	T
6	31229737	rs3130941	C	G
6	31253891	rs3095254	C	G
6	31264334	rs3130542	A	G
6	31267728	rs2853953	G	A
6	31270541	rs9264638	G	A
6	31272654	rs2074488	G	T
6	31273332	rs13191343	C	T
6	31274397	rs2524079	G	A
6	31279426	rs2853946	A	T
6	31284619	rs2524054	A	C
6	31285148	rs12191877	C	T

continued on the next page

Chromosome	Position	dbSNP ID	Reference	Alt allele
6	31291060	rs9468925	G	A
6	31295974	rs2894207	T	C
6	31297713	rs2247056	T	C
6	31303939	rs9461688	A	G
6	31304484	rs6457374	C	T
6	31306603	rs9264942	T	C
6	31306778	rs10484554	C	T
6	31344549	rs3134792	T	G
6	31354782	rs2523608	G	A
6	31355013	rs2523607	T	A
6	31356323	rs148203517	G	T
6	31359287	rs2523590	T	C
6	31359924	rs9378249	T	G
6	31364962	rs9266359	C	T
6	31368124	rs2922994	A	G
6	31368641	rs9266406	G	A
6	31379045	rs9266629	T	C
6	31379674	rs2244020	A	G
6	31382927	rs1521	C	T
6	31384336	rs9266772	T	C
6	31385552	rs2596565	G	A
6	31394533	rs16899524	C	T
6	31398818	rs2596542	C	T
6	31412752	rs2256183	A	G
6	31422633	rs2516448	T	C
6	31440488	rs2524276	C	A
6	31462150	rs3094228	T	C
6	31466334	rs3094604	A	G
6	31472892	rs3828890	C	G
6	31479019	rs2248462	G	A
6	31481199	rs3099844	C	A
6	31504943	rs2855812	G	T
6	31507709	rs3132468	C	T
6	31513522	rs2516399	A	G
6	31537703	rs2734583	A	G
6	31574531	rs1799964	T	C
6	31600692	rs2857595	G	A
6	31605179	rs2844479	A	C
6	31607499	rs9348876	C	T
6	31620607	rs2857693	G	T
6	31623121	rs2736172	C	T
6	31628105	rs1046080	C	A
6	31634066	rs3115663	T	C
6	31635190	rs1046089	G	A
6	31635993	rs9267522	A	G
6	31648589	rs805303	G	A
6	31652743	rs3117582	T	G
6	31654829	rs805297	C	A
6	31664357	rs3130618	C	A
6	31753256	rs3131379	G	A
6	31834764	rs9368699	T	C
6	31870936	rs494620	G	A
6	31876147	rs2736428	C	T
6	31883457	rs652888	A	G
6	31899476	rs9267663	C	T
6	31902549	rs558702	G	A

continued on the next page

Chromosome	Position	dbSNP ID	Reference	Alt allele
6	31903079	rs9267665	C	T
6	31915902	rs9267673	C	T
6	31946403	rs641153	G	A
6	31949174	rs541862	T	C
6	31949763	rs4151657	T	C
6	31962664	rs592229	G	T
6	31973120	rs389884	A	G
6	32051969	rs2857009	G	C
6	32059031	rs12198173	G	A
6	32082290	rs185819	T	C
6	32082981	rs1150754	C	T
6	32102292	rs41268896	G	A
6	32103240	rs3117181	C	G
6	32107027	rs12153855	T	C
6	32108722	rs2269426	G	A
6	32142202	rs204999	A	G
6	32146738	rs9296009	A	T
6	32159700	rs3134950	C	A
6	32166733	rs3096697	G	A
6	32168770	rs1061808	T	G
6	32178715	rs3134945	C	A
6	32183666	rs2070600	C	T
6	32187804	rs204993	A	G
6	32190542	rs176095	A	G
6	32197667	rs2071278	A	G
6	32203298	rs3132935	A	G
6	32203906	rs2071277	T	C
6	32205216	rs3131296	C	T
6	32212119	rs2071286	C	T
6	32216568	rs404860	T	C
6	32220606	rs422951	T	C
6	32222251	rs3132946	A	G
6	32222629	rs443198	A	G
6	32222843	rs3134931	T	C
6	32224554	rs3096702	A	G
6	32237268	rs549182	G	A
6	32237333	rs9267911	T	C
6	32240547	rs424232	C	T
6	32249315	rs6936204	T	C
6	32249455	rs9267972	A	G
6	32251066	rs3115573	A	G
6	32251212	rs9296015	G	A
6	32255481	rs3130320	T	C
6	32276850	rs3130340	T	C
6	32289789	rs926070	G	A
6	32293475	rs7775397	T	G
6	32315077	rs6910071	A	G
6	32338202	rs3129900	G	T
6	32347950	rs910049	T	C
6	32351860	rs9268301	G	A
6	32368410	rs3129934	T	C
6	32368989	rs3129939	A	G
6	32369853	rs2273017	G	A
6	32370918	rs3129943	A	G
6	32371299	rs2050190	A	G
6	32373576	rs9268402	G	A

continued on the next page

Chromosome	Position	dbSNP ID	Reference	Alt allele
6	32390493	rs3117099	G	A
6	32390736	rs3117098	G	A
6	32395438	rs1980493	T	C
6	32396039	rs2076530	T	C
6	32396067	rs9268480	C	T
6	32396178	rs2076529	T	C
6	32400310	rs3817963	T	C
6	32405921	rs3806156	G	T
6	32408196	rs3763309	C	A
6	32408694	rs3763313	A	C
6	32409011	rs3763317	C	T
6	32411712	rs9268516	C	T
6	32415273	rs4959027	A	G
6	32416944	rs9268542	A	G
6	32420032	rs2395163	T	C
6	32421871	rs3135363	A	G
6	32425204	rs3135350	C	T
6	32433162	rs115306967	G	C
6	32433302	rs3129860	A	G
6	32433440	rs3135338	C	T
6	32438565	rs3129871	A	C
6	32440750	rs9268645	C	G
6	32441753	rs3129882	G	A
6	32443869	rs7192	T	G
6	32444658	rs3177928	G	A
6	32445274	rs3135388	A	G
6	32445682	rs2227139	G	A
6	32445768	rs3129889	G	A
6	32446496	rs3129890	T	C
6	32457105	rs7763262	T	C
6	32460508	rs6903608	C	T
6	32460938	rs114800139	G	A
6	32460995	rs9268839	A	G
6	32461866	rs9268853	T	C
6	32461942	rs9268856	C	A
6	32463370	rs9268877	A	G
6	32464185	rs114002140	A	G
6	32464300	rs9268905	G	C
6	32465058	rs9268923	C	T
6	32465390	rs2395185	G	T
6	32476421	rs12194148	G	T
6	32476767	rs12195582	C	T
6	32478940	rs7453498	T	C
6	32480822	rs7748270	C	T
6	32533367	rs2157337	C	T
6	32553130	rs3828840	T	C
6	32601914	rs477515	G	A
6	32604474	rs2858870	T	C
6	32605694	rs9270965	A	G
6	32606214	rs9270984	T	G
6	32606283	rs9270986	A	C
6	32606394	rs615672	G	C
6	32607881	rs3021304	G	C
6	32608701	rs9271100	T	C
6	32609018	rs9271117	CA	TG
6	32609603	rs660895	A	G

continued on the next page

Chromosome	Position	dbSNP ID	Reference	Alt allele
6	32610753	rs9271192	C	A
6	32615965	rs9271348	G	A
6	32619077	rs9271366	G	A
6	32623148	rs3129763	G	A
6	32623176	rs9271588	T	C
6	32624423	rs9271640	T	C
6	32627446	rs9271858	A	G
6	32632222	rs9272105	G	A
6	32633026	rs9272143	T	C
6	32634492	rs9272219	G	T
6	32635230	rs2040406	A	G
6	32636595	rs9272346	G	A
6	32638107	rs2187668	C	T
6	32638979	rs9272535	G	A
6	32644619	rs6927022	TA	CG
6	32658092	rs9273349	T	C
6	32658353	rs7744020	G	A
6	32658495	rs9273363	C	A
6	32658534	rs6906021	T	C
6	32658824	rs9273373	A	G
6	32659936	rs1063355	GT	AG
6	32659937	rs1063355	T	G
6	32660651	rs2854275	C	A
6	32665055	rs9274407	A	T
6	32665936	rs9274477	A	G
6	32683763	rs17212223	C	T
6	32687441	rs2856683	T	G
6	32689801	rs7774434	T	C
6	32690302	rs7775228	T	C
6	32690533	rs9469220	G	A
6	32692101	rs9275224	A	G
6	32695854	rs3129720	T	C
6	32696074	rs6457617	C	T
6	32696222	rs6457620	G	C
6	32696681	rs2647012	T	C
6	32697183	rs9357152	A	G
6	32698518	rs9275319	A	G
6	32698883	rs9275326	C	T
6	32700133	rs2647044	G	A
6	32700323	rs2647045	G	A
6	32700559	rs2647046	A	C
6	32701379	rs9275390	T	C
6	32702178	rs9275406	G	T
6	32702478	rs2856718	C	T
6	32702531	rs2856717	A	G
6	32707332	rs9275524	T	C
6	32710135	rs9275563	C	T
6	32710820	rs4273729	C	G
6	32711222	rs9275572	A	G
6	32712799	rs7764819	T	G
6	32713151	rs7765379	T	G
6	32713500	rs2858331	A	G
6	32713578	rs9275595	T	C
6	32713854	rs9275596	C	T
6	32713892	rs6935723	T	C
6	32713899	rs3104402	T	G

continued on the next page

Chromosome	Position	dbSNP ID	Reference	Alt allele
6	32714360	rs3957148	A	G
6	32717773	rs3916765	G	A
6	32720196	rs9275698	A	G
6	32730008	rs2859113	C	G
6	32732306	rs2858884	A	C
6	32739518	rs9276370	G	T
6	32756140	rs7756516	C	T
6	32757416	rs2301271	A	G
6	32759026	rs1049110	C	T
6	32762235	rs7453920	A	G
6	32762309	rs2051549	G	A
6	32768918	rs9276606	A	T
6	32774091	rs2621416	T	C
6	32795737	rs2857151	A	G
6	32819259	rs1894407	C	A
6	32830099	rs241436	A	G
6	32836293	rs241428	T	G
6	32842071	rs9357155	G	A
6	32843852	rs2071543	G	T
6	32945469	rs1480380	C	T
6	32985503	rs3097645	G	C
6	33004627	rs3128935	T	C
6	33005822	rs9276975	C	T
6	33007157	rs378352	G	A
6	33007237	rs399604	T	C
6	33080884	rs1042151	A	G
6	33087084	rs9277535	A	G
6	33087761	rs9277554	C	T
6	33087828	rs9277555	G	A
6	33102116	rs3117242	A	G
6	33114531	rs733208	G	A
6	33118472	rs3117035	C	T
6	33118671	rs1883414	G	A
6	33121846	rs3117027	C	A
6	33129837	rs3129269	C	A
6	33176171	rs2254287	C	G
6	33236497	rs9277952	G	A
6	65855460	rs9354308	G	A
6	68275038	rs10455657	T	G
7	40483	rs6583337	G	A
8	1425554	rs28680850	G	A
8	2021565	rs6558578	G	T
8	2165853	rs17685410	A	G
8	2229242	rs4876199	T	G
9	88196267	rs2814828	T	C

GWAS Hits that overlap with ASDPs

Table S5: High-impact ASDPs not listed in dbSNP. The table shows 10 ASDP-associated variants which were annotated in our in-house cohort with putative high impact. The `count` column contains the number of occurrences annotated as ASDP or not annotated. The `info` column offers various annotations separated by '|': gene symbol|RefSeqID|HGVS.cdna|HGVS.protein

chrom	position	ref	alt	counts	scaffold	info
chr2	131794984	A	AT	23/53	KI270768.1	HC2orf27B|NM_214461.2|c.808dup|p.(Ile270Asnfs*4)
chr4	68646933	T	TC	49/119	GL000257.2	UGT2B15|NM_001076.3|c.1763dup|p.(*588Trpext*11)
chr6	30949435	T	C	17/121	GL000250.2	DPCR1|NM_080870.3|c.970T>C|p.(*324Gln)
chr6	32828676	C	CCTCCACCCCA	5/7	GL000250.2	TAP2|NM_000544.3|c.2290_2291insTGGGGTGGAG|p.(Gly764Valfs*29)
chr15	30361719	G	A	1/32	GL383554.1	CHRFAM7A|NM_139320.1|c.1813C>T|p.(Gln605*)
chr15	32603707	C	T	1/65	GL383554.1	GOLGA8N|NM_001282494.1|c.1810C>T|p.(Gln604*)
chr17	37610127	A	AT	4/42	KI270857.1	DDX52|NM_001291476.1|16/16|c.5644dup|p.(Ile1882Asnfs*3)
chr19	54307242	C	CA	4/6	GL949746.1	LILRA5|NM_021250.3|c.1070dup|p.(Leu357Phefs*40)
chr19	54307242	C	CAA	2/3	GL949746.1	LILRA5|NM_021250.3|c.1069_1070dup|p.(Leu357Phefs*2)
chr22	23981036	A	G	4/11	KI270879.1	GSTT2|NR_126445.1|n.370+1A>G|

Algorithm S1 Determine candidate seed

```
Input: A candidate matching (M) block in a pairwise alignment
SVMIN ⇐ 50                                          ▷ Minimum length of seed alignment
seed_len ⇐ calculate_length(M Block)
ref_start ⇐ start_position_in_reference_sequence(M Block)
alt_start ⇐ start_position_in_alt_hap_sequence(M Block)
if seed_len * 0.9 < SVMIN then
    output null;                                    ▷ skip this M block because it is too short
end if
CHOP ⇐ floor(seed_len * 0.05)   ▷ Length of sequence to be removed from both ends of the M Block
if CHOP > SVMIN then
    CHOP ⇐ SVMIN                                    ▷ Do not remove more than SVMIN residues
end if
ref_start ⇐ ref_start + CHOP
alt_start ⇐ alt_start + CHOP
seed_len ⇐ seed_len − (2 * CHOP)
Output: Seed alignment starting at position ref_start in the reference sequence and alt_start
in the alternate haplotype sequence, and having a length of seed_len (Note that this block of the
alignment has no gaps).
```

Algorithm S2 ASDPex

Input: Region $\mathcal{R}$, list of ASDPs associated with $\mathcal{R}$, Sample genotype data $\mathcal{G}$ (e.g., from VCF file)
A ⇐ Variants called in sample in region $\mathcal{R}$
B ⇐ ASDPs in region $\mathcal{R}$
RV ⇐ $A \triangle B$ ▷ residual variants
if $|\mathtt{A}| < |\mathtt{RV}|$ **then**
    haplotype inferred ***homozygous REF***
else
    hom ⇐ count_number_of_homozygous_ASPD_associated_variants()
    N ⇐ count_total_number_of_ASPD_associated_variants()
    if $\mathtt{hom}/N > 0.9$ **then**
        haplotype inferred ***homozygous ALT***
    else
        haplotype inferred ***heterozygous ALT***
    end if
end if
Output: Inference on most likely genotype for region $\mathcal{R}$ (homozygous REF, homozygous ALT scaffold, heterozygous). Mark appropriate variants as ASPD unless genotype called as homozygous REF.

References

[1] Gotoh, O.: An improved algorithm for matching biological sequences. J Mol Biol **162**(3), 705–708 (1982)

[2] Brudno, M., Do, C.B., Cooper, G.M., Kim, M.F., Davydov, E., , N.I.S.C.C.S.P., Green, E.D., Sidow, A., Batzoglou, S.: Lagan and multi-lagan: efficient tools for large-scale multiple alignment of genomic dna. Genome Res **13**(4), 721–731 (2003). doi:10.1101/gr.926603

[3] Jäger, M., Wang, K., Bauer, S., Smedley, D., Krawitz, P., Robinson, P.N.: Jannovar: a java library for exome annotation. Hum Mutat **35**(5), 548–555 (2014). doi:10.1002/humu.22531

[4] Eilbeck, K., Lewis, S.E.: Sequence ontology annotation guide. Comp Funct Genomics **5**(8), 642–647 (2004). doi:10.1002/cfg.446
